# ‘Social’ versus ‘asocial’ cells—dynamic competition flux balance analysis

**DOI:** 10.1038/s41540-023-00313-5

**Published:** 2023-10-28

**Authors:** Yanhua Liu, Hans V. Westerhoff

**Affiliations:** 1 Swammerdam Institute for Life Sciences, University of Amsterdam, Amsterdam, The Netherlands; 2 Molecular Cell Biology, A-Life, Faculty of Science, Amsterdam Institute for Molecules, Medicines and Systems, Vrije Universiteit Amsterdam, Amsterdam, The Netherlands; 3 School of Biological Sciences, Faculty of Biology, Medicine and Health, University of Manchester, Manchester, UK; 4 Stellenbosch Institute for Advanced Study (STIAS), Wallenberg Research Centre at Stellenbosch University, Stellenbosch, 7600 South Africa

**Keywords:** Systems biology, Computational biology and bioinformatics

## Abstract

In multicellular organisms cells compete for resources or growth factors. If any one cell type wins, the co-existence of diverse cell types disappears. Existing dynamic Flux Balance Analysis (dFBA) does not accommodate changes in cell density caused by competition. Therefore we here develop ‘dynamic competition Flux Balance Analysis’ (dcFBA). With total biomass synthesis as objective, lower-growth-yield cells were outcompeted even when cells synthesized mutually required nutrients. Signal transduction between cells established co-existence, which suggests that such ‘socialness’ is required for multicellularity. Whilst mutants with increased specific growth rate did not outgrow the other cell types, loss of social characteristics did enable a mutant to outgrow the other cells. We discuss that ‘asocialness’ rather than enhanced growth rates, i.e., a reduced sensitivity to regulatory factors rather than enhanced growth rates, may characterize cancer cells and organisms causing ecological blooms. Therapies reinforcing cross-regulation may therefore be more effective than those targeting replication rates.

## Introduction

The cells in a multicellular organism or in a stable ecosystem have different *inherent* specific growth and turnover rates. Peripheral T and B cells are renewed for 30~40% every 48 h^1^, red blood cells every 120 days^2^ and brain cells rarely^3^. ‘Cell competition’ was first mentioned in a study of ‘*Minute*’ mutants in Drosophila’s cell division rate^4^. These mutants grew more slowly, resulting in their elimination. Cell-cell competition for limited nutrients, growth factors, or space can optimize tissue fitness by eliminating ill-functioning cells through apoptosis^5^. Such competition can also be exploited however by super-competitor cells, e.g., high Myc expression cells outcompeting cells expressing little Myc^6^.

Cell-cell competition may involve both metabolism and signal transduction, with signaling pathways regulating growth, apoptosis, engulfment and interaction between winner and loser cells^7,8^. Competition between immune and tumor cells is an emerging hallmark of tumors^9^. Additionally, tumor and host cells may compete for metabolic resources^10^. Conversely, the lactic acid secreted by many tumor cells may inhibit neighboring cells or immune cells^11,12^, while ammonium secretion may do this for tumors with the WarburQ phenotype^13^.

Understanding how cell-cell competition and intercellular communication affect the stability of populations of cells of different cell types, might help develop adjuvant therapies for diseases where the balances between different cell types are disturbed. These include autoimmune diseases, hyperplasia and cancer^14^. Imbalance between the human body and its microbiomes may lead to additional diseases^15^.

Cells compete for nutrition such as glucose, glutamine and oxygen. These compounds contribute to the cells’ growth processes (e.g., ATP and biomass synthesis or lactic acid secretion)^11,12^. Cells may also help each other. Lung cells help provide heart cells with oxygen and heart cells help lung cells through circulation, for instance. Yet, heart cells cannot grow as much as they might ‘want’ because their excessive growth would diminish nutrition for lung cells resulting in insufficient oxygen availability for the heart cells. How cells make the necessary ‘informed’ choices in cell communities where metabolism is involved, and what such informed choices are, is incompletely understood. Flux Balance Analysis (FBA)^16^ has become the method of choice for calculating balanced metabolism in complex networks. With some modifications it can simulate metabolism by integrating gene mutation and nutrient concentration information^17^, and identify drug targets^18^. However, to a system with multiple cell types competing for nutrition, standard FBA is not directly applicable as cell numbers and thereby balanced metabolic fluxes vary over time. *Dynamic* FBA (dFBA) allows fluxes to vary with time at time scales longer than time scales required for metabolic relaxation inside the cells. dFBA typically uses kinetic equations for dominant nutrient supply rates as functions only of concentrations of growth substrates outside the network. This may work well for single cell type growth^19^ or co-cultures on multiple nutrients^20^. However, it addresses neither the impact of cell number variation with time^21^ nor intercellular competition for nutrients. The cell concentrations should be dependent on the dynamic nutrient concentrations. Conversely the total nutrient uptake rate for each cell type should be affected by cell concentrations. Whilst detailed kinetic modelling^22,23^ could address metabolic networks with time variant cell numbers and competition, it requires extensive kinetic details that are largely unknown. It lacks the much-reduced requirement of kinetic details that FBA offers.

This paper develops a form of FBA that deals with competition between different cell types for nutrients as well as with cross regulation of the same cells through signal transduction. The new variant of FBA, called dynamic competition FBA (dcFBA), integrates the cells’ competition for nutrients with the cells’ growth. These two factors are made mutually dependent and variable. Cell number values calculated at each time point are subsequently employed to modulate the nutrient uptake rates, effectively representing cell-cell competition. Applying this dcFBA we elucidate the intricate behavior of diverse cell types within a multicellular system, as they rely on intercellular metabolites or growth factors for sustenance. We explore strategies to achieve system stability. Moreover, the new dcFBA is harnessed to simulate the behavior of ‘asocial’ cells and potential therapies controlling such cells.

## Results

### Neither competition for common substrate nor metabolic cross-talk produces stable coexistence in standard FBA in a two-cell types system

**Fig. 1:**
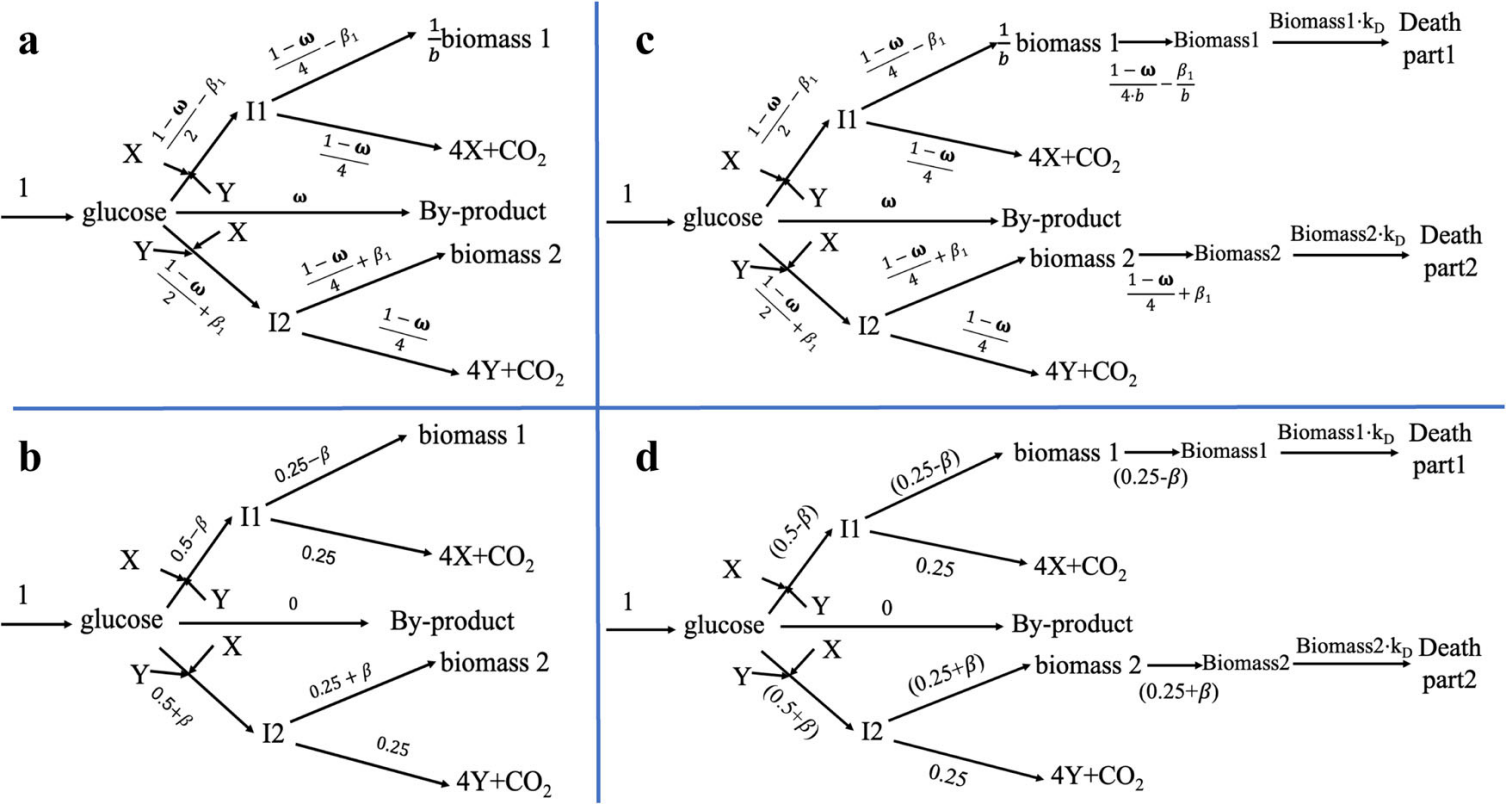
Network structures of two cell types. Metabolic (**a**, **b**) and Growth-metabolic (**c**, **d**) network structures. The two cell types compete for a common substrate and depend on common goods X and Y. Stoichiometries equal 1 except for the synthesis of 1/*b* C-mol biomass 1 from 1 C-mol of I1 and the producing of 4 molecules of X and Y from their respective precursors I1 and I2. Balanced flux values (in C-mol/time unit) are written above or below the reaction arrows. In (**a**) and (**c**), ω can assume any positive value smaller than 1, whilst the growth bias *β*_*1*_ can assume any value between -$\frac{1-\omega }{4}$ and + $\frac{1-\omega }{4}$. In the optimal states (**b**, **d**) ω equals 0 and the growth bias is confined to a value that depends on the biomass stoichiometry b of biomass 1. In (**a**): the metabolic network in which CO_2_ and biomass of both cell types constitute the output, whilst glucose, I1, I2, X, Y are balanced metabolites. In (**b**): the *optimal* balanced flux pattern through the network of a with *b* = 1: ω, the flux to by-product, equals zero. The optimal β can assume any value between −0.25 and +0.25. In (**c**): The metabolism-plus-growth network, with fluxes balancing both around the metabolites (glucose, I1, I2, X, Y) and the biomass levels (biomass1 and biomass2). In (**d**): As (**c**) but with optimal balanced flux values indicated: Again, *ω* = 0 and for *b* = 1, the optimal β can assume any value between −0.25 and +0.25. Otherwise *β* = −0.25 (if *b* < 1) or +0.25 (for *b* > 1). For *b* = 1, the glucose consumption bias is equal to the biomass synthesis bias (i.e., *β* = *β*_*1*_).

We first examined whether two cell types that compete for a common metabolic substrate can reach steady coexistence. The answer is “No” (Supplementary Fig. 1 and Supplementary Table 1, Supplementary Fig. 2 and Supplementary Table 2). In the case of Supplementary Fig. 3 where there is no competition for substrate, both cell types can grow exponentially. The metabolic network (Fig. 1) allows for the two cell types also to depend on each other through ‘common goods’ X (produced by cell type 1) and Y (produced by cell type 2) that both cell types require for their synthesis of X or Y and their growth (Fig. 1a). Some glucose may escape to by-product at flux rate ω. The scheme has 8 reactions, 5 metabolic intermediates (glucose, I1, I2, X, and Y) and one fixed flux (the glucose influx). A flux balance analysis (i.e., requiring steady state for the 5 intermediates) produced two modes of variation of the system. We selected the flux to by-product and the difference between the biomass synthesis rates of cell type 2 and cell type 1 (which we called β1 in Fig. 1a and β in Fig. 1b) as variables to monitor these modes (Fig. 1a). With total biomass synthesis as objective function, the flux to by-product dropped to zero (Fig. 1b). Sections 1-3 of the supplementary results show that the optimal flux always ran either to the ‘cheapest biomass’ (i.e. the biomass with the highest growth yield) or to both cell types with different growth fluxes (Supplementary Fig. 5 and Supplementary Table 3, Supplementary Fig. 6 and Supplementary Table 4). Competition for a common metabolic substrate and interdependence through common goods such as in Fig. 1a does not suffice for coexistence of cell types (also at stoichiometries of X and Y synthesis different from 4 (Supplementary Note 3, Supplementary Figs. 7, 8)).

Figure 1c, d remind us that the actual network structure should also require balances around the concentrations of biomass 1 and biomass 2 (section 3-1 of Supplementary Results). These additions did not really affect to outcome of Fig. 1b however: in the corresponding Fig. 1d the growth rate of the cheapest cell type still became persistently higher than the specific growth rate of the more ‘expensive’ cell type. Consequently, no coexistence arose.

Figure 1c lacks an aspect of reality, however. If the biomass synthesis flux of cell type 1 is lower than that of cell type 2 whilst the sum of the two fluxes must equal the maximum 0.5, the required synthesis rates of X and Y remain 1. The amount of biomass of type 1 will however decrease with time. As time goes on, the same amount of X has to be synthesized by less and less of cell type 1. The FBA that we used up to this point neither accommodates a maximum to the X synthesis per unit biomass of cell type 1 nor that the growth cell type 2 should then stop, perhaps causing coexistence. In the next sections we develop a variant of the FBA that is able to address these issues.

### Stepwise growth FBA for two-cell types system

We developed a ‘stepwise-growth FBA’ algorithm whereby at each time point the rate of biomass synthesis is related to the cell number. The points in Supplementary Figs. 11a–c display the predicted cell numbers as functions of time for three growth rate biases *β.* Again, one cell type ultimately outcompeted the other cell type at any growth bias. Paradoxically, as cell type 1 with its capacity to synthesize the common good X disappeared, cell type 2 continued to grow even though a shortage of X should arise. The required capacity for X synthesis, i.e., the flux per unit cell 1, increased to infinity (red line in Supplementary Figs. 11d–f).

**Fig. 2:**
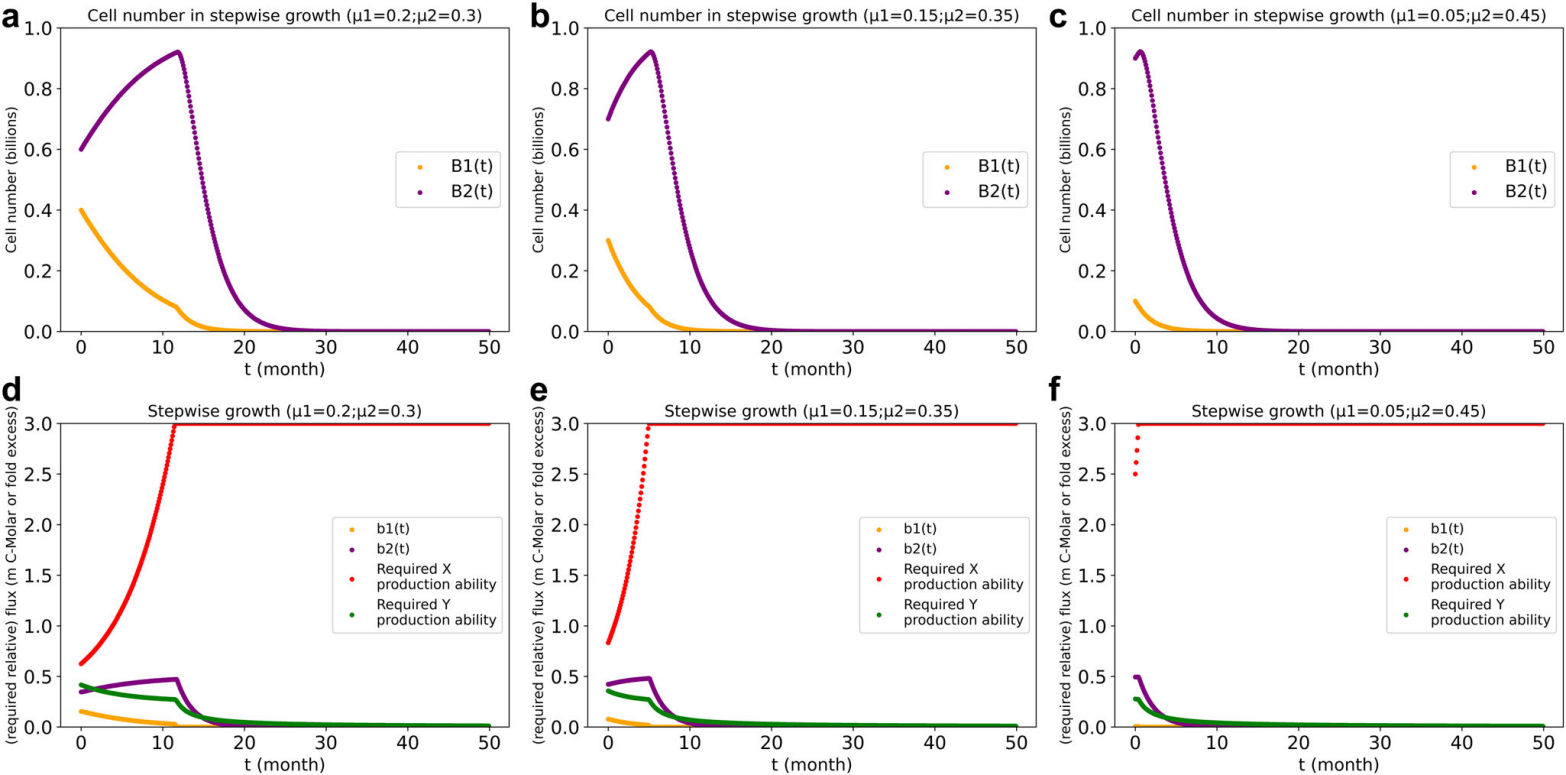
Biomass levels (B1, B2) and related biomass synthesis rate (b1 and b2) and the required X and Y production for cell type 1 and cell type 2, respectively. **a**, **d**: *β* = 0.05; (**b**, **e**): *β* = 0.1; (**c**, **f**): *β* = 0.2. The Biomass levels and related biomass synthesis rates are calculated with capacities of metabolic reactions (I1 to X and I2 to Y) limited to 3 times the corresponding Biomass level, as calculated using the stepwise-growth FBA algorithm as described under Materials and Methods. Glucose influx was unlimited and glucose efflux absent. The FBA objective was maximal total biomass synthesis, calculated by adding the balance between b1 and b2 and the death rate (Biomass 1 or Biomass 2 respectively, multiplied by *k*_*D*_ = 0.5/*ts*) after every time step of 0.1 month. The volume of the culture vessel was assumed to be 1 Liter (same in all calculations in the paper).

We then set maximum production capacities of X and Y per unit biomass of cell types 1 and 2 (as specified under Methods). Initially, Biomass 2 again increased whilst Biomass 1 decreased and with it its upper bound for X synthesis. After t = 12 months (for *β*(0) = 0.05, Fig. 2a), the upper bound for X synthesis became lower than what was required. Biomass 2 abruptly decreased with time and the decrease with time of Biomass 1 accelerated. Total biomass thereby also decreased with time, ultimately to zero. A greater growth bias difference led to a shorter lifespan (i.e., a shorter duration of community co-existence). Using the stepwise growth algorithm with maximized metabolic capacities still no stable coexistence was obtained thereby. The actual growth rate bias ${\beta }(t)=\frac{{\rm{b}}2\left({\rm{t}}\right)-{\rm{b}}1({\rm{t}})}{2}$ changed with time to zero (Supplementary Fig. 12). An exponential growth model (see Supplementary Figs. 9, 10) and a kinetic model integrated by Copasi^24^ (Supplementary Fig. 13) did not reach coexistence either. Only less realistic cases (e.g., $\frac{{\mu }_{1}}{{k}_{D1}}=\frac{{\mu }_{2}}{{k}_{D2}}$ or ‘time variant death rate constants’) could lead to coexistence (Supplementary Figs. 14, 17).

### Stepwise growth FBA with cross regulation does show stability

**Fig. 3:**
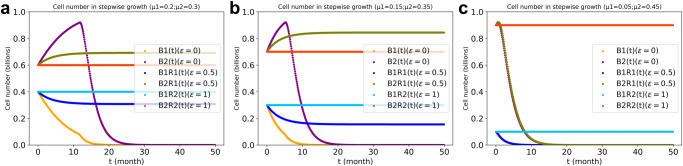
Stepwise growth of the two cell types system at various magnitudes of the regulation power *ε* and growth rate bias β. $\beta \stackrel{\scriptscriptstyle\mathrm{def}}\,{=}\,\left({\mu }_{2}-{\mu }_{1}\right)/2$. In (**a**): $\beta$ = 0.05; (**b**): $\beta$ = 0.1; (**c**): $\beta$ = 0.2; all three calculations with capacities of metabolic reactions limited as in Fig. 2. The value of $\varepsilon$ indicates the strength of the regulation power ($\varepsilon$ = 0: without regulation). The purple and orange points show the biomass value for cell type 2 and cell type 1, respectively, without regulation. The light-blue and olive points show the biomass value for cell type 1 and type 2, respectively, with regulation power of 0.5. The deep-sky-blue points and orange-red points are for the case with regulation power of 1, for cell type 1 and cell type 2, respectively. The stepwise-growth-with-regulation FBA algorithm was used with total biomass production as objective function.

Now we introduce direct cross-regulation between the two cell types. The biomass synthesis rate of cell type 1 $\left({b}_{1,s,{r\; ub}}(t)\right)$ will now be considered to be positively regulated (ε > 0, ε is the ‘elasticity coefficient’ of the regulation^25^) by the number of cells of type 2 as represented by a factor ${\left({B}_{2,s,r}\left(t\right)\right)}^{\varepsilon }$ in the numerator (the consequent negative dependence produces the required carbon flux balance, i.e., the competition for the glucose). Cell coexistence arose when the regulation power was strong enough (0.5 or 1 in Fig. 3). The purple and yellow lines recall that in the absence of such regulation ($\varepsilon =0$) no such stable coexistence developed. The minimum regulation power ($\varepsilon$ value) for coexistence at three initial growth biases was 0.17 (for *β*(0) = 0.05), 0.36 (for *β*(0) = 0.1) and 0.92 (for *β*(0) = 0.2).

In the presence of the regulation, the steady state was reached before the maximum capacity was hit. Consequently, the metabolic limitation was now irrelevant for coexistence. Complete regulation, i.e., an elasticity coefficient ε = 1, sufficed to bring the two cell types into coexistence even if their inherent growth rates differed by a factor of 9. At a high inherent growth bias, stable co-existence required a strong regulation power (Fig. 3c). In the kinetic model, co-existence could also be achieved when such regulation was put in (see Supplementary Fig. 16), but again not for the exponential growth model (Supplementary Fig. 15).

### An asocial mutant in a stable two-cell types stepwise growth system

**Fig. 4:**
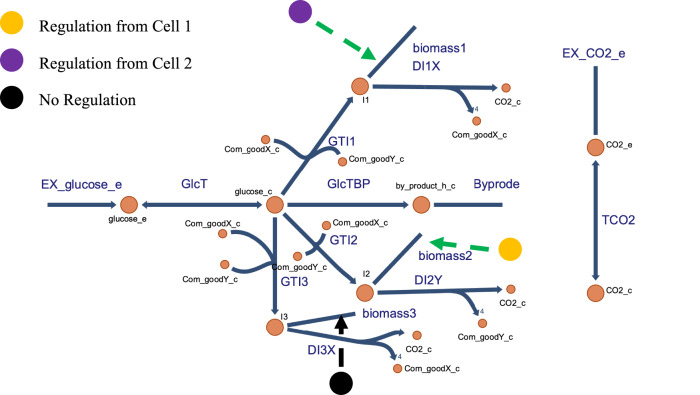
The three cell types’ network. Cell type 3 is a mutant of cell type 1 just losing the regulation by and of cell type 2, and is taken to exemplify an ‘asocial cell’ as it does not engage in cross regulation. All three cell types equally require X and Y for growth. Cell types 1 and 3 both produce X whilst cell type 2 produces Y.

We then introduced a third cell type, again under consideration of growth optimization for total biomass synthesis, flux balance, regulation, and limited metabolic capacity (Fig. 4). We modeled the third cell type as a mutant of cell type 1 that continues to use common goods X and Y and converts glucose to its intermediate I3, and that produces X but has undergone a mutation interfering with the cross regulation with cell type 2. As a control we first considered a cell type 3 that was the same as type 1, i.e., neither ‘non-responsive’ nor ‘non-communicating’ (respectively meaning that the mutant is not responsive to regulation by cell type 2, or does not regulate cell type 2). The results (Supplementary Fig. 18) were the same as in Fig. 3a with regulation power of 1. Then we considered three cases for the new mutant: (1) both non-responsive and non-communicating; (2) non-responsive; (3) non-communicating.

**Fig. 5:**
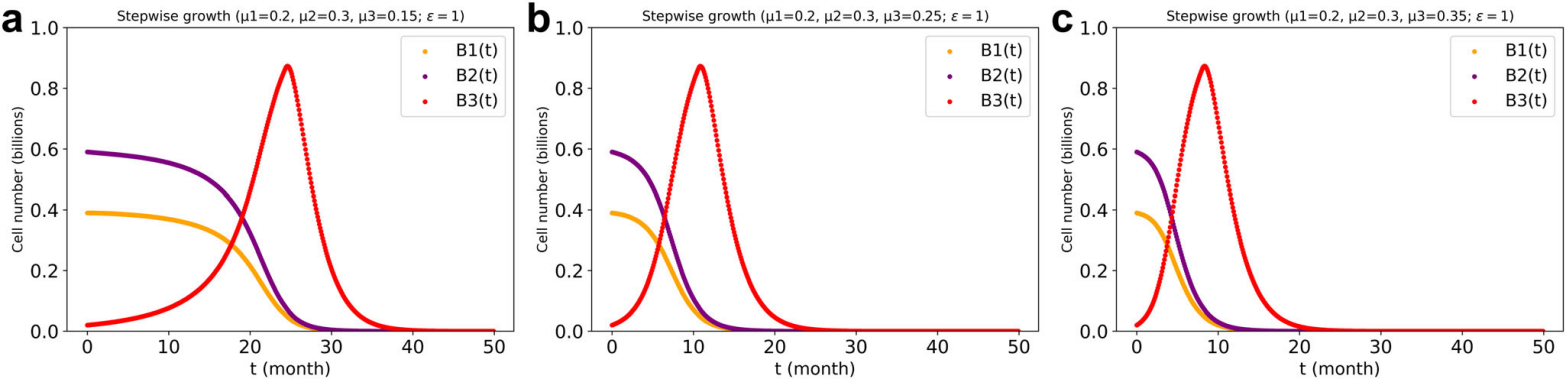
Cell numbers of the three cell types with various differences between their inherent specific growth rates in the three cell types’ system. The reciprocal regulation between the ‘social’ cell types 1 and 2 had an elasticity of 1, (i.e., $\varepsilon$ = 1). The third cell type was both ‘non-responsive’, i.e., insensitive to such regulation, and ‘non-communicating’, i.e., did not regulate either other cell types. In (**a**): a lower inherent specific growth rate for the asocial cell type 3 (i.e., ${\mu }_{3}\,$ < ${\mu }_{1}$ < ${\mu }_{2}$). In (b): an intermediate inherent specific growth rate for the asocial cell (i.e., ${\mu }_{1}\,$ < ${\mu }_{3}$ < ${\mu }_{2}$). In (**c**): a higher inherent specific growth rate for the asocial cell (i.e., ${\mu }_{1}\,$ < ${\mu }_{2}$ < ${\mu }_{3}$). Initial cell numbers were 0.39, 0.59, and 0.02 billion for cell types 1, 2, and 3, respectively. The stepwise growth FBA algorithm for the three cell types’ system with cross-regulation elasticity (*ε*) of 1 between cell type 1 and type 2 was used for the dcFBA computations with total biomass synthesis as objective function and with the metabolite production capacity limitation present. If no cell type 3 was added, the simulation was identical to that of Fig. 3a ($\varepsilon$ = 1).

In case 1 the mutant cell type 3 outgrew the other two cell types, ultimately bringing all cell numbers down to zero. The transient outgrowth of the mutant cells occurred even though it had the same (or lower) inherent specific growth rate (0.2/*ts*) as cell type 1 and an even lower inherent specific growth rate than cell type 2 had (Supplementary Fig. 19 and Fig. 5). In the ‘unresponsiveness’ case 2, the mutant cell type 3 co-existed with cell type 2, but outgrew cell type 1 (Supplementary Fig. 20). Since the mutant (cell type 3) has the same functionality as its original (cell type 1), it did not make the system unstable. In the ‘no communication’ case 3 the mutant could not outgrow the other two cell types and only existed at low concentrations. This may simulate a benign tumor (Supplementary Fig. 21). That (see case 1) in a system comprising of social cell types, the ‘asocial’ mutant should outgrow the other cell types, might seem remarkable because the signals it stopped responding to, appear to be stimulatory. An increase in the number of type 2 cells was modelled as enhancing the growth of the cell so that after the mutation, this stimulation was lost, suggesting that the mutant should be worse and not better off. That the result is the opposite is due to the growth regulating factor being smaller than 1 and proportional to cell numbers in order to control excessive cell growth.

### Three cell types regulating each other in the three-cell types system

**Fig. 6:**
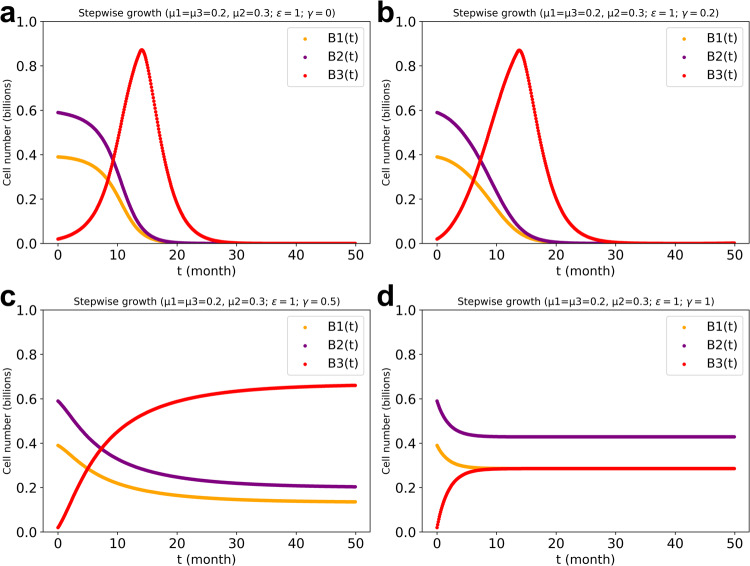
Cell numbers developing over time in the three cell types’ system with various regulation powers (*γ*) from normal cell types to type 3 and vice versa. **a**: $\gamma =0$ (no such cross regulation). **b**: $\gamma =0.2$. **c**: $\gamma =0.5.$ **d**: $\gamma =1.$ The regulation power between cell type 1 and cell type 2 was taken $\varepsilon =1$ reciprocally. The inherent growth rate bias in favor of cell type 2 as compared to the other two cell types was taken to equal $\beta =0.05$. The stepwise growth FBA algorithm for three cell types’ system with cross-regulation power of 1 (i.e., $\varepsilon =1$) between cell type 1 and type 2 and also regulation among three cell types, was used for the dcFBA computations with total biomass synthesis as objective function and with the production capacity limitation present.

The emergence of the transformed cell type that has lost all regulation through mutation is unlikely. Most breast cancers are still estrogen dependent for instance^26,27^. We therefore also considered the situation in which all three cell types continue to regulate each other at various elasticities. When we increased the reciprocal cross-regulation elasticity (*γ* value) between the normal cells of type 1 and type 2 on the one hand and the ‘transformed’ cell type 3 on the other hand we found that already at the moderate regulation of *γ* = 0.5 cell type 3 no longer outgrew the other two cell types (Fig. 6). For an initial population composition of B_1_(0) = 0.39, B_2_(0) = 0.59 and B_3_(0) = 0.02, the minimal magnitude of *γ* for stability was around 0.3 at a death rate of 0.5/*ts*. Additionally, we investigated the higher growth rate of cell type 3 with and without regulation by the other two cell types. Cell type 3 thrived at a high growth rate when regulation by the other two cell types was active, leading to the observed coexistence (Supplementary Fig. 22d). Also this result shows that cell regulation rather than inherent growth rate, is the factor determining whether cells out-compete each other or co-exist (This was confirmed by kinetic modelling (Supplementary Figs. 25, 26)).

### Only cell type 3 (mutant) being regulated by the other two cell types (normal cells)

**Fig. 7:**
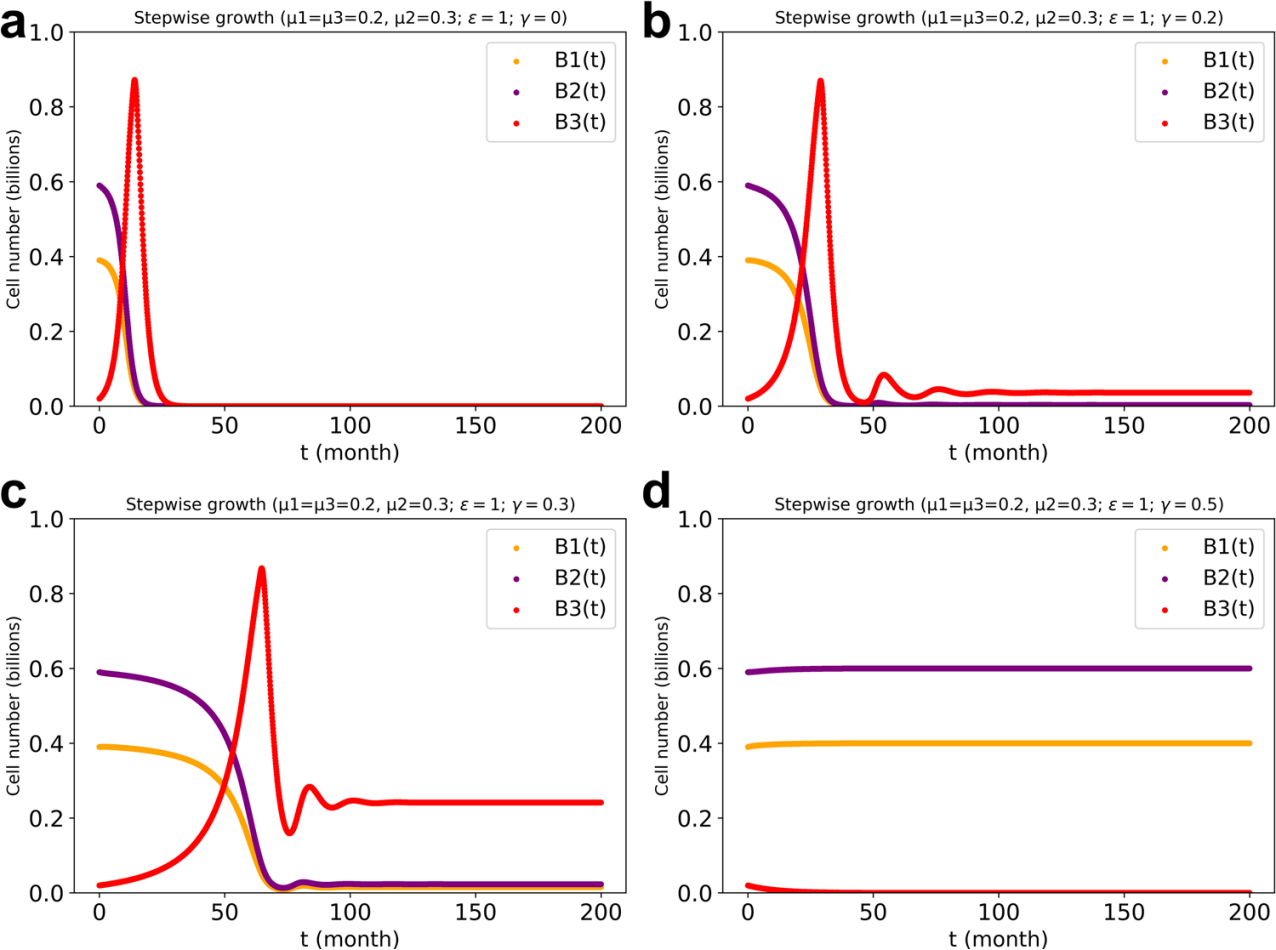
Cell numbers in the three cell types’ system with various regulation powers (i.e., various *γ* values) between cell type 3 and the other two cell types. **a**: $\gamma =0$ (no such cross regulation). **b**: $\gamma =0.2$. **c**: $\gamma =0.3.$ **d**: $\gamma =0.4.$ The inherent growth rate bias in favor of cell type 2 as compared to the other two cell types was taken to equal $\beta =0.05$. We calculated over a longer time (i.e., t = 200 months) to show the steady state. The stepwise growth algorithm in the three cell types’ system with cross-regulation power of 1 (i.e., $\varepsilon =1$) between cell type 1 and type 2 and also only regulation to the third cell type from the other two cell types was used for the stepwise-growth FBA computations with maximal total biomass synthesis as objective and with maximum metabolic production capacity in place.

In actual situations the transformed cell is still reliant on the normal cells’ activities. However, the tumor cell may not support the normal cells in any way. When increasing the cross-regulation (*γ* value) between the normal cells and the transformed cell, the transformed cell initially outgrew the other two cell types, but this could subsequently lead to coexistence of the three cell types (Fig. 7c). However, this coexistence is not a typical occurrence since more often the normal cell count drastically decreased to zero. As the regulation strength was increased to 0.4 or more, the two normal cell types outgrew the transformed cell (as shown in Fig. 7d).

### Evaluation of therapeutic strategies based on model simulations: specific cytotoxic treatments or therapies aimed at restoring regulation

**Fig. 8:**
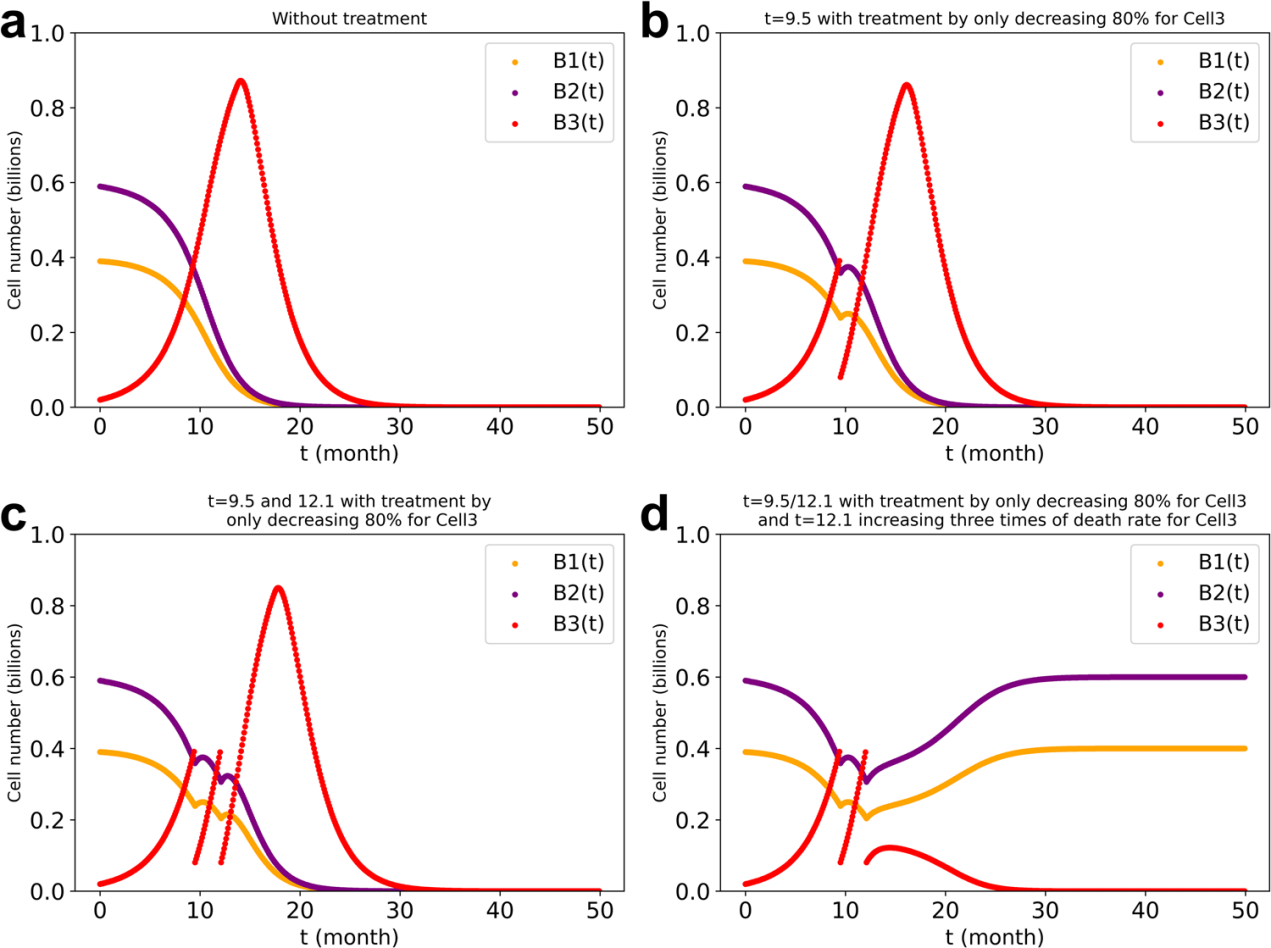
Simulated therapy of decreasing only the tumor cell number by 80% when it reaches a critical value 0.4 billion. **a**: no treatment. **b**: single instantaneous treatment at time 9.5 months. **c**: dual instantaneous treatment at times 9.5 and 12.1 months. **d**: the same as (**c**) but also increasing the death rate constant of cell type 3 at *t* = 12.1 months by a persistent factor of 3. The stepwise growth algorithm in the three cell types’ system with cross-regulation elasticity of 1 between cell type 1 and type 2 and no regulation of cell type 3 by the other two cell types was used for the stepwise-growth dcFBA computations with maximal total biomass synthesis as objective. ${\mu }_{1}\,$ = ${\mu }_{3}=0.2;{\mu }_{2}=0.3$. The different values of ‘*t*’ refer to the time of treatment.

In the perspective that cell type 1 and cell type 2 represent normal cells, and cell type 3 asocial (tumor or ecological bloom) cells, we simulated potential therapies. We next modelled three interacting cell types (1, 2 and 3) starting at initial cell numbers of 0.39, 0.59 and 0.02 billion, respectively. The inherent specific growth rates of cell type 1 and 3 were taken equal at 0.2/*ts* and that of cell type 2 was taken 0.3/*ts*, with the same specific death rate of 0.05/*ts* for all three. We chose a critical value of 0.4 billion for the tumor cell number and simulated the therapy by reducing the tumor cell number by 80% at the first (Fig. 8b) or at the first two critical time points (Fig. 8c, d). Our results showed that continued therapy (i.e., a persistent decreasing of the number of the tumor cells) should be necessary to keep the number of tumor cells from again reaching the critical value and from causing the system to become unstable (which we attributed to cells of type 1 or 2 dwindling to 0.1 billion (Fig. 8)). Although instantaneous therapies reduced the number of tumor cells by 80%, they only increased the time at which the system became unstable by approximately 10%.

However, by exposing tumor antigens this therapy has the potential to activate the immune systems and increase the concentration of antitumor T cells, which presumably outreaches the suppression of immune inhibitory pathways by the tumor cells. We simulated this by also tripling the death rate constant of the tumor cells once their number had decreased to such a low value (i.e., just after the second therapeutic intervention (Fig. 8d)). The results confirm that as adjuvant to immune therapy this side effect of killing tumor cells might be effective whereas it would not be in the absence of the immune therapy (see however ref. ^28^).

**Fig. 9:**
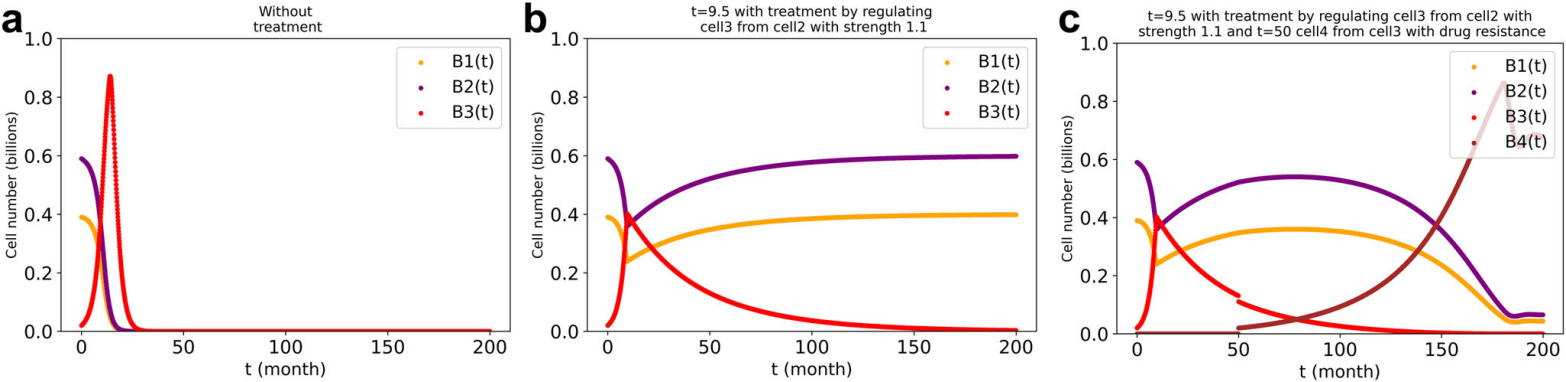
Simulated therapy of restoring the regulation (to elasticity = 1.1) of the tumor cells by cell type 2 when the tumor cell number attains a critical level (i.e., 0.4 billion), and also the drug resistance of tumor cells (i.e., the brown line, cell type 4). **a**: without treatment. **b**: treatment at *t* = 9.5. **c**: treatments at *t* = 9.5 and *t* = 50, the latter time point arising drug resistance (brown line: cell type 4 is the same as the tumor cell (i.e., the red line, cell type 3) but with drug resistance). The stepwise-growth FBA algorithm in the three cell types’ system with cross-regulation elasticity of 1 between cell type 1 and type 2 and no regulation of cell type 3 by the other two cell types was used for the stepwise-growth dcFBA computations with maximal total biomass synthesis rate as objective. ${(\mu }_{1}\,$ = ${\mu }_{3}=0.2;{\mu }_{2}=0.3$). ‘*t*’ means the treatment time.

Next, we investigated the effects of a particular drug or therapy that restored the regulation to cell type 3 by type 2. The simulation assumed that the drug would increase the elasticity of cell type 3 with respect to regulation by cell type 2 (i.e., ε_1_) from 0 (without therapy) to 1.1 with therapy. We chose 1.1 because the number for cell type 3 was dominant when therapy was needed. Accordingly, the regulated strength should be higher than the elasticity between cell types 1 and 2. At a critical tumor cell number of 0.4 billion the therapy could prevent the system from becoming unstable due to tumor cells increasing in its cell number (Fig. 9b). Similarly, our results indicated that continuous therapies would still be required to maintain the stability of system unless the regulation could stay in place forever after one or a few therapies. The development of drug resistance in tumor cells might still lead to a gradual loss of efficacy, however. The brown line in Fig. 9c shows the newly drug resistant tumor cells increasing, whilst the original tumor cell was disappearing (the red line).

## Discussion

A new, ‘dynamic competitive FBA’ (dcFBA) methodology was developed and used to investigate the behavior of a multicellular system over time. Mathematical equations set the reaction bounds for a nutritionally competitive situation. The biomass values for each cell type were incorporated into the nutrient uptake equations. Additionally, the biomass synthesis was balanced to account for cell number growth, wherein the biomass synthesis was determined by competitive nutrient uptake. Small time intervals were employed to iterate dynamic progress and the flux balance requirement. The interplay among biomass synthesis, cell number increase and nutrient uptake competition, governed the growth of cell populations and influenced overall dynamics. Comparing with dFBA, this new dcFBA method not only shows how cell growth relates to competition for nutrients, but also puts cell numbers into the nutrient uptake rate equation to show competition mathematically. The dcFBA could also be used to simulate interactions between cells through ‘growth factors’. For two cell types with different inherent growth rates, regulation was necessary to prevent one cell type from going extinct. In a three-cell types system involving two normal cell types and a transformed cell type, the loss of regulation led to the emergence of ‘asocial’ cells that exploited resources from others. By using dcFBA to simulate the persistence and growth of such asocial cells, we showed that this asocialness and not a higher intrinsic growth rate was responsible for the mutant outgrowing the normal cells and for endangering the multicellular organism. Although this result has been discussed^29–31^ previously, this is the first time its feasibility has been demonstrated (and in this sense ‘explained’) by using mathematical equations set in a relatively simple modelling approach (i.e., dcFBA) and by assuming an objective of cell proliferation (i.e., biomass synthesis, see below). Furthermore, dcFBA was applied to simulating drug treatments by decreasing the cell number of tumor cells. We did not only show that continuous therapy would be needed, but also that this may prove ineffective due to the development of drug resistance. Alternative therapeutic interventions restoring regulation in tumor cells would also necessitate ongoing treatment. Nevertheless, the latter approach should hold the potential of maintaining regulatory control over the offspring of the tumor cells.

While the current system comprises only a limited number of reactions, the obtained results will be similar when using more complex metabolic maps to represent the different cell types (see the supplementary material). The incorporation of additional nutrients through such maps leads to increased nutrient competition ultimately impacting biomass production and nutrients uptake, which is similar to the findings presented here (Supplementary Fig. 29). For understanding the full and detailed complexity of ecosystems other modelling methods^32,33^, perhaps in combination with dcFBA, should also be of great interest. dcFBA developed here may also be useful for other issues around tissue homeostasis, such as the control of liver size. Within minutes after partial hepatectomy, mammalian liver exhibits regenerative abilities, recovering to its original organ mass within weeks^34,35^. The liver is also capable of returning to its normal size after hypertrophy and/or hyperplasia, by the activation of regression mechanisms (e.g., cell apoptosis), using mechanisms monitoring cell number or cell size^36^. One may implement the dcFBA developed here to try and describe this precise regulation and the reason why there is no overgrowth. Another example may reside in the competition between astrocytes and neurons for amino acid uptake during brain development. In a previous paper^37^, we developed another mode of competitive FBA to demonstrate implications of the amino acid competition for uptake across the blood-brain barrier. One may now fruitfully combine the two new types of FBA and include neurotransmitter cross-talk and metabolism. FBA differs from kinetic modelling in requiring virtually no kinetic detail^15^. Since it is these kinetic details that are still missing for the complex networks at hand, this should make the dcFBA approach a useful addition to the modelling toolbox, at least for the not-too-distant future.

FBA’s prediction of much fewer than all possible steady state behaviors, requires the assumption that network behavior is optimal, however. The optimality is usually assumed to correspond to maximal growth rate of the cells in question^16^ and that is what we also assumed here: maximality of *total* biomass synthesis. However, one could try various objective functions (as Supplementary Figs. 27, 28) and utilize a more suitable and precise objective function for the specific FBA calculation as our previous paper did, for a different circumstance^37^. In multicellular organisms the specific growth rate of the various cell types is known to be controlled tightly. We could have used this as starting point, for instance by taking the specific growth rate of any one cell type (perhaps ovary cells or pluripotent stem cells) as objective function under the *proviso* that the specific growth rates of all other cell types should be precisely the same (this in order to prevent any one cell type from overgrowing all the others because it has the highest specific growth rate). Instead we used the total biomass synthesis rate as objective. And we examined under what condition of cross regulation we obtained ‘stability’ (effectively defined as the persistence of all cell types eventually at the same specific growth rate), so that the relative numbers of the cells of the different types would remain time independent. In section 10 of the supplementary material we used the biomass synthesis rate of only one cell type as objective function and obtained essentially the same results. A most important implication is that our paper found an explanation for what we should otherwise have used as a priori, i.e., that all specific growth rates are equal due to tight control. We found that irrespective of the objective being total biomass or the biomass of only one of the cell types stability required such tight control. Another implication is that we found the appropriate objective function for dcFBA.

Our study did not deliver detail. The integration of more molecular, signaling and interaction information into FBA-kinetics hybrid models may lead to a more realistic representation of metabolic and signaling phenomena. Myc-mediated cell competition for instance is prevalent across different organs and tissues, including fibroblasts^38^ and heart cells^39,40^ and plays a role in the growth and expansion of cancer cells^6,41–43^. Overexpression of Myc transforms tumor cells into super-competitors, enabling them to eliminate adjacent wild-type cells^44^. The growth signaling pathway in out-competed cells becomes impaired^5^, as evidenced by the decreased decapentaplegic transduction observed in out-competed cells^45^: the out-competed cells capture fewer growth factors. The winner cells increase their engulfment^46^, thereby acquiring more space and resources while evading regulatory mechanisms from normal cells. Tumor cells also inactivate the Hippo pathway, thereby promoting their own proliferation^47^. The dcFBA developed here may be extended to deal with some of this complexity.

Competition for nutrients, space, and growth factors is ubiquitous in multicellular organisms. On the one hand, normal or young cells compete with damaged or older cells to maintain tissue homeostasis. Preventing such competition may result in lymphoblastic leukemia^48^. On the other hand, when asocial tumor or super-competitor cells engage in cell competition, normal cells may be eliminated. Our findings suggest that the critical time (i.e., the time it takes for the tumor to grow to a critical size) can be prolonged by increasing the initial cell number of normal cells (cell type 1 and type 2) (Supplementary Fig. 23). This may imply that an active body at a reduced glucose (metabolic substrate) level might delay the growth of tumor cells, suggesting an alternative to glucose fasting^14^. That also the cell-death rate constant affected the critical time (Supplementary Fig. 24) suggests that elder individuals may be more sensitive to competition by tumor cells. Our results may further inspire the development of drugs or therapies targeting the competition or cross-regulation, rather than cytotoxic agents or growth rate inhibitors. A higher inherent specific growth rate is unlikely to be the primary factor driving tumor cell growth (Fig. 5 and Supplementary Fig. 22). When social cells with a higher inherent specific growth rate were present, the asocial cell type (cell type 3) was able to outgrow them but not when the latter was social even if it had a *higher* specific growth rate than the other cell types.

In this paper we employed mathematical equations to establish the biomass synthesis balance within a meta-metabolic map (i.e., a map involving more than one organism albeit in a drastically simplified form), effectively illustrating cellular competition by adjusting the cells’ nutrient uptake rates based on their respective cell numbers. By applying this novel methodology we successfully simulated a community comprising multiple cell types and elucidated the critical role played by intercellular regulation in the achievement of system stability. This approach may hold potential for investigating complex microbial ecosystems, effectively showcasing their intricate dynamics in terms of competition and cooperation. It could provide a comprehensive understanding of cellular interactions within a community, shedding light on the factors that govern stability and cooperation in intricate biological systems through mathematical equations.

Our observations of the effects of cell-cell competition and cross-regulation may also be relevant more generally. Also, the components of an ecosystem are interdependent and single ‘dominant’ species or mutants rarely thrive in isolation. Relevant scenarios in global warming or human-induced pollution may be analyzed by our new dcFBA methodology with a proper objective function and network.

## Methods

### Model building—two cell types competing for common free-energy/carbon substrate and interacting through common goods

In all our models two (or three) cell types use glucose to produce their own metabolic intermediate (I1 and I2, respectively) (Supplementary Fig. 4). The so-called ‘common goods’ X and Y are produced by cell type 1 and 2 from their respective metabolic intermediates and are both required by both cell types to produce their metabolic intermediate. Each cell type uses its intermediate either to produce its own biomass (i.e., to increase its cell number) or to produce one common good. The model also contains a by-product reaction from glucose and a carbon dioxide secreting reaction. In terms of carbon, our models transform half the glucose into biomass and the other half into carbon dioxide, a carbon/carbon yield that is not unusual^49^. The stoichiometries for the GTI1 (glucose-to-intermediate-1) reaction (see Supplementary Fig. 4) are taken to be -1 for glucose, X, and Y and +1 for I1. For the GTI2 reaction they are analogously -1 for glucose, X and Y and +1 for I2. For DI1X they are -1, +4, and +1 for I1, X, and CO_2_, respectively. For DI2Y they are -1, +4, and +1 for I2, Y, and CO_2_, respectively. For the reaction towards biomass 1 they are -1 and +1 (or 1/b) for the intermediate I1 and the biomass 1, respectively. For synthesis of biomass 2, these numbers are -1 and +1 for the intermediate I2 and the biomass 2, respectively. In our simple model, only glucose was supplied in the medium. This model may correspond to the interaction between ‘lung cells’ and ‘liver cells’, which both depend for their growth on externally supplied glucose (for carbon), glutamine (for nitrogen) and oxygen (for energetics) in the circulating blood. Lung cells oxygenate the blood, whilst liver cells enrich it with glutamine. Both cell types may use the glucose, glutamine and oxygen in growth and respiration producing carbon dioxide and urea.

By using the COBRA routine^50,51^, FBA was performed with the sum of the two biomass production fluxes as objective and the maximum total biomass synthesis flux was determined. This produced flux balances for glucose, X and Y, as well as I1 and I2. Acknowledging that COBRA’s FBA only yields a single flux pattern also when there are multiple equivalent patterns, we employed Flux Variability Analysis (FVA)^52^ to compute the range of possible flux patterns at the same maximum magnitude of the objective function, by limiting each step to smaller absolute values. We also checked the effect of making biomass 1 ‘more expensive’ by making the production of biomass1 cost 2 units of I1, and biomass 2 ‘cheaper’ by requiring only one unit of I2 to produce 1 unit of biomass 2. In other models the biomass 1 synthesis reaction only produce 1/*b* C-molar of biomass per C-molar of I1 (The volume of the culture vessel was assumed to be 1 Liter for all calculations in this paper).

### Growth FBA—dynamic cell competition flux balance analysis for two cell types competing for a common substrate and with time varying cell densities, without or with capacity limitations, and with cross dependence through common goods X and Y

According to the FBA the biomass synthesis fluxes should optimally add up to 0.5 (This was because we assumed the glucose uptake equaled 1. In cases where the glucose uptake differs from this value, the equations should be adjusted). Assuming that this optimum is achieved at all times, also as the biomass concentration ratios change, the biomass synthesis fluxes for biomass 1 and biomass 2, respectively, should tend towards Eqs. (1) and (2).

1 $${b}_{1,e,{ub}}(t)=0.5\cdot \frac{{\mu }_{1}\cdot {f}_{1}(t)}{{\mu }_{1}\cdot {f}_{1}(t)+{\mu }_{2}{\cdot f}_{2}(t)}$$

2 $${b}_{2,e,{ub}}(t)=0.5\cdot \frac{{\mu }_{2}\cdot {f}_{2}(t)}{{\mu }_{1}\cdot {f}_{1}(t)+{\mu }_{2}{\cdot f}_{2}(t)}$$

with arbitrary functions *f*_*1*_*(t)* and *f*_*2*_*(t)* representing the growth tendencies, and with ${\mu }_{1}$ ${\rm{and}}$ ${\mu }_{2}$ representing the inherent specific growth rates.

#### Stepwise growth FBA for two cell types

We first assumed pre-defined exponential functions of time for two growth tendencies, and found that one cell type number reached 0 when time tended to infinity (see Supplementary Material). We next developed an FBA growth algorithm in which the growth kinetics would not be pre-defined. At the glucose branch we considered fluxes towards I1, I2 and an overflow flux ${\rm{\omega }}$, the rates of which we assumed to be all proportional to the glucose concentration level. The biomass synthesis ratios at the next time (t+ts, *ts* is a small (infinitesimal) amount of time) was taken proportional to their cell number at the time t $\left(\frac{{b}_{1,s,{ub}}(t+{ts})}{{b}_{2,s,{ub}}(t+{ts})}=\frac{{\mu }_{1}\cdot {B}_{1,s}\left(t\right)}{{\mu }_{2}\cdot {B}_{2,s}\left(t\right)}\right)$. This was described as differing in *μ*, but this could just as well reflect a difference in yield. With the common goods balance met and the glucose influx fixed to 1, the two fluxes towards biomass amounted to Eqs. (3) and (4).

3 $${b}_{1,s,{ub}}(t+{ts})=\frac{{0.5\cdot \mu }_{1}\cdot {B}_{1,s}\left(t\right)\cdot \left(1-{\rm{\omega }}\right)}{{\mu }_{1}\cdot {B}_{1,s}\left(t\right)+{\mu }_{2}\cdot {B}_{2,s}\left(t\right)}$$

4 $${b}_{2,s,{ub}}(t+{ts})=\frac{{0.5\cdot \mu }_{2}\cdot {B}_{2,s}\left(t\right)\cdot \left(1-{\rm{\omega }}\right)}{{\mu }_{1}\cdot {B}_{1,s}\left(t\right)+{\mu }_{2}\cdot {B}_{2,s}\left(t\right)}$$

Because the growth rates were reset at every time point of computation, we called this the ‘stepwise-growth FBA’ procedure. The rate of X synthesis required was equal to twice the sum of these two fluxes. Hence the fluxes from glucose to I1 and I2 are described by Eqs. (5) and (6).

5 $${v}_{1,g}(t+{ts})=\frac{1-{\rm{\omega }}}{4}+\frac{{0.5\cdot \mu }_{1}\cdot {B}_{1,s}\left(t\right)\cdot \left(1-{\rm{\omega }}\right)}{{\mu }_{1}\cdot {B}_{1,s}\left(t\right)+{\mu }_{2}\cdot {B}_{2,s}\left(t\right)}$$

6 $${v}_{2,g}(t+{ts})=\frac{1-{\rm{\omega }}}{4}+\frac{{0.5\cdot \mu }_{2}\cdot {B}_{2,s}\left(t\right)\cdot \left(1-{\rm{\omega }}\right)}{{\mu }_{1}\cdot {B}_{1,s}\left(t\right)+{\mu }_{2}\cdot {B}_{2,s}\left(t\right)}$$

Total biomass synthesis is described by Eq. (7).

7 $${b}_{1,s,{ub}}(t)+{b}_{2,s,{ub}}(t)=\frac{1-{\rm{\omega }}}{2}$$

When asking for maximal total biomass synthesis, and if the metabolic capacities were not limited, ${\rm{\omega }}$ became equal to zero and disappeared from the equations. At every time point FBA was carried out for the metabolic network of Supplementary Fig. 4, with *b*_*1,s,ub*_ and *b*_*2,s,ub*_ as the upper bounds for the biomass synthesis rates for cell types 1 and 2, respectively. This produced the biomass synthesis fluxes $b_{1,s,FBA}(t)$ and $b_{2,s,FBA}(t)$, which were effectively equal to the upper bounds. Acknowledging that the cells should also be subject to death processes (for which we used a first order process with rate constant *k*_*D*_ (we chose *k*_*D*_ to equal 0.05/*ts*)), we calculated the Biomass concentrations for the two cell types at each time point from Eqs. (8) and (9).

8 $${B}_{1,s}\left(t+{ts}\right)={B}_{1,s}\left(t\right)\cdot \left(1-{ts}\cdot {k}_{D}\right)+{ts}\cdot {b}_{1,s,FBA}\left(t\right)$$

9 $${B}_{2,s}\left(t+{ts}\right)={B}_{2,s}\left(t\right)\cdot \left(1-{ts}\cdot {k}_{D}\right)+{ts}\cdot {b}_{2,s,FBA}\left(t\right)$$

where *ts* = 0.1 month; B_1_(0) = 0.4 and B_2_(0) = 0.6 for *β* = 0.05; B_1_(0) = 0.3 and B_2_(0) = 0.7 for *β* = 0.1; B_1_(0) = 0.1 and B_2_(0) = 0.9 for *β* = 0.2 (These values were the same for subsequent calculations unless specifically stated). We chose these values because we wanted the whole system to be stable before it became unstable (new biomass flux equaling the death flux). The difference between the cell numbers at the beginning was in accordance with their relative growth rates.

#### Limiting metabolic capacities

The FBA calculations using the methods specified up to this point did not acknowledge any possible capacity limitation for producing common goods X or Y that would arise due to lack of sufficient cells of type 1 or 2, respectively. The model continued to predict biomass synthesis of cell type 2 even when cell type 1 (the sole producer of the X required by cell type 2 for the synthesis of its intermediate I2 and thereby for its growth) had been outgrown. To address this issue, we adjusted the upper bound for X and Y production so as to be proportional to the cell numbers of type 1 and type 2, respectively. Specifically, we set the maximum abilities to produce X or Y to three times their respective calculated cell numbers. After setting the reaction bound, we again ran the FBA at every time point to compute the growth rates of two cell types (i.e., $b_{1,s,FBA}(t)$ and $b_{2,s,FBA}(t)$). By the usual multiplication of the biomass production fluxes by the duration of the time step and by correction for cell death, we then calculated the predicted cell numbers for each cell type. In an alternative methodology, 1- ${\rm{\omega }}$ in the Eq. (7) was replaced according to Eq. (10).

10 $$1-{\rm{\omega }}(t+{ts})={minimum}(1{\rm{;}}\,{4\cdot {V}_{\max }\cdot B}_{1}\left(t\right){\rm{;}}\,{4\cdot {V}_{\max }\cdot B}_{2}\left(t\right))$$

We also developed a kinetic model based on irreversible mass action rate equations and used Copasi^24^ to simulate the cells’ growth dynamics (See Supplementary Material, Supplementary Tables 5, 6).

### Regulation in stepwise growth FBA

We also considered cases where the two cell types depended on each other for their growth also more directly than through the common goods X and Y. We assumed that cell type 2 produced a growth factor that stimulated growth of cell type 1 without being consumed by it, and that the concentration of that growth factor was proportional to the number of cells of type 2. The regulation is assumed to depend on the activation of a receptor by the binding of growth factor G, as described by Eq. (11).

11 $${\rm{G}}+{\rm{R}}\leftrightharpoons {\rm{RG}}$$

Here G is the growth factor produced by cell type 2 and R is the corresponding receptor in the plasma membrane of cell type 1. At binding equilibrium is described by Eq. (12).

12 $${\rm{RF}}=\frac{[{\rm{RG}}]}{\left[{\rm{R}}\right]+[{RG}]}=\frac{[{\rm{G}}]}{[{\rm{G}}]+{K}_{d}},$$

where the regulation factor (RF) is the fraction of receptor bound to growth factor G; *K*_*d*_ is the dissociation equilibrium constant. If α is the ratio of the production rate constant to the first-order dilution rate constant of G, then:

13 $${\rm{G}}={\rm{\alpha }}\cdot {B}_{2}$$

14 $${\rm{RF}}=\frac{{\rm{\alpha }}\cdot {B}_{2}}{{\rm{\alpha }}\cdot {B}_{2}+{K}_{d}}=\frac{{B}_{2}}{{B}_{2}+\frac{{K}_{d}}{{\rm{\alpha }}}}$$

The regulation factor will always be smaller than 1 and depend on B_2_. For simplicity we used B_2_ (the number of cells of cell type 2) to represent the activity factor which then acts at various different strengths (We also tried using various values of $\frac{{K}_{d}}{{\rm{\alpha }}}$ (e.g. 0.2, 1 and 5); this led to similar results as compared to when we chose the number of cells of type 2 as regulation factor (see: Supplementary Fig. 30)). We ensured that B_2_ ranged between 0 and 1, by adjusting the unit for cell numbers (i.e., to a billion).

To simulate the regulation we made the rate of synthesis of cell type 1 proportional to RF (and hence to the concentration of cell type 2) taken to the power of the ‘elasticity’ $\varepsilon$ (as described in Eq. (15)).

15 $${b}_{1,s,r,{ub}}\left(t+{ts}\right)=\frac{0.5{\cdot \mu }_{1}\cdot \left({B}_{1,s,r}\left(t\right)\right)\cdot {\left({B}_{2,s,r}\left(t\right)\right)}^{\varepsilon }}{{\mu }_{1}\cdot \left({B}_{1,s,r}\left(t\right)\right)\cdot {\left({B}_{2,s,r}\left(t\right)\right)}^{\varepsilon }+{\mu }_{2}\cdot {\left({B}_{1,s,r}\left(t\right)\right)}^{\varepsilon }\cdot \left({B}_{2,s,r}\left(t\right)\right)}$$

$\varepsilon$ is the elasticity coefficient of the regulation^25^ akin to the power in Biochemical Systems Theory^53^. The corresponding dependence of synthesis of cell type 2 on the concentration of cell type 1 reads as Eq. (16).

16 $${b}_{2,s,r,{ub}}(t+{ts})=\frac{0.5{\cdot \mu }_{2}\cdot {\left({B}_{1,s,r}\left(t\right)\right)}^{\varepsilon }\cdot \left({B}_{2,s,r}\left(t\right)\right)}{{\mu }_{1}\cdot \left({B}_{1,s,r}\left(t\right)\right)\cdot {\left({B}_{2,s,r}\left(t\right)\right)}^{\varepsilon }+{\mu }_{2}\cdot {\left({B}_{1,s,r}\left(t\right)\right)}^{\varepsilon }\cdot \left({B}_{2,s,r}\left(t\right)\right)}$$

Here *b*_*1,s,r,ub*_ and *b*_*2,s,r,ub*_ were again used to set the upper bounds for the biomass synthesis reactions. After that we obtained actual biomass synthesis rates for cell types 1 and 2 with regulation in stepwise growth, respectively. And we used these values to calculate the cell number for cell types 1 and 2 by Eqs. (8) and (9). Although in the beginning the inherent specific growth rates for cell types 1 and 2 were *μ*_*1*_ = 0.25-$\beta$ and *μ*_*2*_ = 0.25+$\beta$, respectively, the two actual specific growth rate tendencies would depend on time. We tried various regulation powers (i.e., different values of $\varepsilon$) to set the reaction upper bound for biomass production and to check how the cell number was changing with time. As the equation does not contain a predefined *exponential* growth tendency for the two cell types but this growth tendency is set by the stepwise change in biomass concentrations with time, we call this procedure ‘regulated stepwise growth FBA’.

### Three cell types

#### Stepwise growth FBA for two social cell types together with one asocial cell type

After calculating the system with two cell types, we added another cell type to the system. The third cell type was a mutant of cell type 1 deficient in the communication with cell type 2. Otherwise it functioned identically to cell type 1. It still had the ability to produce common good X, and used common goods X and Y whilst converting glucose to its intermediate metabolite I3. The model building file is provided in the folder ‘files’ in the GitHub directory. The influx was again 1 C-mole/*ts*, the sum of biomass 1, biomass 2 and biomass 3 synthesis was again 0.5 C-mole/*ts*. The excess carbon produced in the reactions forming X and Y was supposed to leave the system as CO_2_. We wanted to examine whether there could be coexistence of the three cell types. We first just studied the control case (i.e., $\partial$ = 1 and $\varepsilon 1=\varepsilon ,$ Eqs. (17) and (18)) in which cell type 3 was the same as cell type 1, i.e., with regulation to cell type 2 and responsive to cell type 2 regulation. Then, we considered three ‘asocial’ cases, i.e., case 1 (‘non-responsive and non-communicating’, i.e., cell type 3 neither regulating/stimulating cell type 2, nor responsive to cell type 2 regulation, i.e., $\partial$ = 0 and $\varepsilon 1=0$), case 2 (‘non-responsive’, i.e., cell type 3 not regulated by cell type 2, cell type 2 still regulated by cell type 3), i.e., $\partial$ = 1 and $\varepsilon 1=0$, respectively. We also considered the remaining case 3 (‘non-communicating’, i.e., cell type 3 not regulating cell type 2, but still regulated by cell type 2, i.e., $\partial$ = 0 and $\varepsilon 1=\varepsilon$). With the common goods balance met and the glucose influx fixed to 1, the three maximal fluxes towards biomass amounted calculation were as described in Eqs. (17), (18) and (19).

17 $${b}_{1,s3,{ub}}\left(t+{ts}\right) = \frac{0.5\cdot {\mu }_{1}\cdot {{B}_{1,s3}\left(t\right)\cdot \left({B}_{2,s3}\left(t\right)\right)}^{\varepsilon }}{{\mu }_{1}\cdot {{B}_{1,s3}\left(t\right)\cdot \left({B}_{2,s3}\left(t\right)\right)}^{\varepsilon }+{\mu }_{2}\cdot {B}_{2,s3}\left(t\right){\,\cdot \,\left({B}_{1,s3}\left(t\right)+\partial \cdot {B}_{3,s3}\left(t\right)\right)}^{\varepsilon }+{\mu }_{3}\cdot {B}_{3,s3}\left(t\right){\,\cdot\, \left({B}_{2,s3}\left(t\right)\right)}^{\varepsilon 1}}$$

18 $${b}_{2,s3,{ub}}\left(t+{ts}\right) = \frac{0.5\cdot {\mu }_{2}\cdot {B}_{2,s3}\left(t\right){\cdot \left({B}_{1,s3}\left(t\right)+\partial \cdot {B}_{3,s3}\left(t\right)\right)}^{\varepsilon }}{{\mu }_{1}\cdot {{B}_{1,s3}\left(t\right)\cdot \left({B}_{2,s3}\left(t\right)\right)}^{\varepsilon }+{\mu }_{2}\cdot {B}_{2,s3}\left(t\right){\,\cdot\, \left({B}_{1,s3}\left(t\right)+\partial \cdot {B}_{3,s3}\left(t\right)\right)}^{\varepsilon }+{\mu }_{3}\cdot {B}_{3,s3}\left(t\right){\,\cdot\, \left({B}_{2,s3}\left(t\right)\right)}^{\varepsilon 1}}$$

19 $${b}_{3,s3,{ub}}(t+{ts}) = \frac{{0.5\cdot \mu }_{3}\cdot {B}_{3,s3}\left(t\right){\cdot \left({B}_{2,s3}\left(t\right)\right)}^{\varepsilon 1}}{{\mu }_{1}\cdot {{B}_{1,s3}\left(t\right)\cdot \left({B}_{2,s3}\left(t\right)\right)}^{\varepsilon }+{\mu }_{2}\cdot {B}_{2,s3}\left(t\right){\,\cdot\, \left({B}_{1,s3}\left(t\right)+\partial \cdot {B}_{3,s3}\left(t\right)\right)}^{\varepsilon }+{\mu }_{3}\cdot {B}_{3,s3}\left(t\right){\,\cdot\, \left({B}_{2,s3}\left(t\right)\right)}^{\varepsilon 1}}$$

Here ‘$\varepsilon$’ or ‘$\varepsilon 1$’ is the inter-regulation between cell type 1 or cell type 3 with type 2 in this ‘three-cell-types’ stepwise growth. Unless specified otherwise the inherent specific growth rates of cell types 1 and 3 were the same (${\mu }_{1}={\mu }_{3}=0.2/{ts}$), while that of cell type 2 was higher (${\mu }_{2}=0.3/{ts}$).

As per the numerators of the Eqs. (17), (18) and (19) we tried various values of $\varepsilon$ and used these equations to set the upper bound for the biomass synthesis rate for the three cell types. The sum of the biomass synthesis rates of the three cell types was again the objective function. After defining the model, we calculated the relationship between the cell number and time by using COBRA at every time step and then integrating over time by the Eqs. (17), (18) and (19) for the Biomasses. Finally, we selected case 1 (both non-responsive and non-communicating) for further analysis because in that case cell type 3 was most similar in behavior to tumor cells.

#### Three cell types regulating each other

We could not find a coexistence for three cell types when we chose various $\varepsilon$ values, even though cell type 3 needed support from the other two in terms of common goods X and Y. As cell type 3 modelled a tumor cell, we considered it unlikely that it had completely lost all regulation. Therefore, we next considered the regulatory interactions among three cell types, whereby cell types 1 and 2 mutually regulated each other with strength (elasticity^25,53^) ε, and were both cross-regulating with cell type 3, and vice versa, with a regulation strength (elasticity) *γ*. The regulation is described in Eqs. (20), (21), (22) and (23).

20 $$\begin{array}{l}{FR}1={\mu }_{1}\cdot {{B}_{1,s3,r1}\left(t\right)\cdot \left({B}_{2,s3,r1}\left(t\right)\right)}^{\varepsilon }{\,\cdot\, \left({B}_{3,s3,r1}\left(t\right)\right)}^{\gamma }\\ \qquad\quad +\,{\mu }_{2}\cdot {\left({B}_{1,s3,r1}\left(t\right)\right)}^{\varepsilon }\cdot {B}_{2,s3,r1}\left(t\right){\,\cdot\, \left({B}_{3,s3,r1}\left(t\right)\right)}^{\gamma }\\ \qquad\quad +\,{\mu }_{3}{\cdot \left({B}_{1,s3,r1}\left(t\right)\right)}^{\gamma }{\,\cdot\, \left({B}_{2,s3,r1}\left(t\right)\right)}^{\gamma }\cdot {B}_{3,s3,r1}\left(t\right)\end{array}$$

21 $${b}_{1,s3,r1,{ub}}\left(t+{ts}\right)=\frac{{0.5\cdot \mu }_{1}\cdot {{B}_{1,s3,r1}\left(t\right)\cdot \left({B}_{2,s3,r1}\left(t\right)\right)}^{\varepsilon }{\,\cdot\, \left({B}_{3,s3,r1}\left(t\right)\right)}^{\gamma }}{{FR}1}$$

22 $${b}_{2,s3,r1,{ub}}\left(t+{ts}\right)=\frac{{0.5\cdot \mu }_{2}\cdot {\left({B}_{1,s3,r1}\left(t\right)\right)}^{\varepsilon }{\,\cdot\, {B}_{2,s3,r1}\left(t\right)\cdot \left({B}_{3,s3,r1}\left(t\right)\right)}^{\gamma }}{{FR}1}$$

23 $${b}_{3,s3,r1,{ub}}(t+{ts})=\frac{0.5\cdot {\mu }_{3}{\,\cdot\, \left({B}_{1,s3,r1}\left(t\right)\right)}^{\gamma }{\cdot \left({B}_{2,s3,r1}\left(t\right)\right)}^{\gamma }\cdot {B}_{3,s3,r1}\left(t\right)}{{FR}1}$$

where ‘$\gamma$’ is the regulation power (elasticity) between the normal cells and cell type 3, and vice versa. ${b}_{1,s3,r1,{ub}}$, ${b}_{2,s3,r1,{ub}}$ and ${b}_{3,s3,r1,{ub}}$ were again used to set the upper bounds for the biomass synthesis reactions. After that, we obtained the actual biomass synthesis rates for cell types 1, 2 and 3 in this three-cell-types system with regulation, by implementing dcFBA at each time point with the sum of their biomass synthesis rates as objective function. These rates were then used for the calculation of the cell numbers.

#### Only cell type 3 (transformed cell) regulated by the two other cell types (normal cells)

In actual situations, the transformed cell needs support from normal cells, but may not support the normal cells in any way. Accordingly, we constructed another regulation network through equations in which the normal cells regulated the transformed cells, and not vice versa. The regulated equations are described by Eqs. (24), (25), (26) and (27).

24 $$\begin{array}{l}{FR}2 = {\mu }_{1}\cdot {{B}_{1,s3,r1}\left(t\right)\cdot \left({B}_{2,s3,r1}\left(t\right)\right)}^{\varepsilon }+{\mu }_{2}\cdot {\left({B}_{1,s3,r1}\left(t\right)\right)}^{\varepsilon }\cdot {B}_{2,s3,r1}\left(t\right)+{\mu }_{3}{\cdot \left({B}_{1,s3,r1}\left(t\right)\right)}^{\gamma }{\cdot \left({B}_{2,s3,r1}\left(t\right)\right)}^{\gamma }\cdot {B}_{3,s3,r1}\left(t\right)\end{array}$$

25 $${b}_{1,s3,r2,{ub}}\left(t+{ts}\right)=\frac{{0.5\cdot \mu }_{1}\cdot {{B}_{1,s3,r1}\left(t\right)\cdot \left({B}_{2,s3,r1}\left(t\right)\right)}^{\varepsilon }}{{FR}2}$$

26 $${b}_{2,s3,r2,{ub}}\left(t+{ts}\right)=\frac{{0.5\cdot \mu }_{2}\cdot {\left({B}_{1,s3,r1}\left(t\right)\right)}^{\varepsilon }\cdot {B}_{2,s3,r1}\left(t\right)}{{FR}2}$$

27 $${b}_{3,s3,r2,{ub}}(t+{ts})=\frac{{0.5\cdot \mu }_{3}{\cdot \left({B}_{1,s3,r1}\left(t\right)\right)}^{\gamma }{\cdot \left({B}_{2,s3,r1}\left(t\right)\right)}^{\gamma }\cdot {B}_{3,s3,r1}\left(t\right)}{{FR}2}$$

Again, we used Eqs. (25), (26) and (27) to set the upper bounds for the biomass production reactions and performed the dcFBA calculation with the sum of the biomass synthesis rates of the three cell types as objective function. And we used the biomass synthesis values obtained by the dcFBA to calculate the cell numbers as in Eqs. (8) and (9).

### Reporting summary

Further information on research design is available in the Nature Research Reporting Summary linked to this article.

### Supplementary information

Supplementary Material---‘Social’ versus ‘Asocial’ cells--- Dynamic Competition Flux Balance Analysis

Reporting Summary

## Data Availability

All data used for the simulations are presented in this manuscript and its Supplementary files.

## Code availability

The code of simulated models is available at Github: https://github.com/YanhuaLiu1/Cells-Competition.
