# Supplementary material for: ‘Social’ versus ‘asocial’ cells—dynamic competition flux balance analysis
Source: NPJ Syst Biol Appl. 2023 Oct 28;9:53. doi: 10.1038/s41540-023-00313-5 (PMC10613221; doi:10.1038/s41540-023-00313-5)
Supplement: Supplementary file 1 — Supplementary Material---‘Social’ versus ‘Asocial’ cells--- Dynamic Competition Flux Balance Analysis [file 41540_2023_313_MOESM1_ESM.pdf]

Supplementary Material

Supplementary Figures

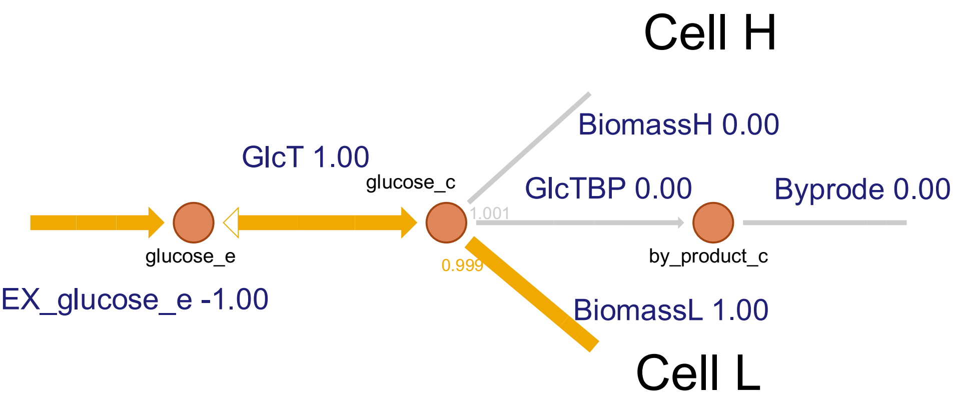

Supplementary Figure 1. Flux balance predicted for cell types 1 (‘Biomass H’) and 2 (‘Biomass L’) competing for a single carbon/free-energy source. An example could be heart cells (‘H’) and lung cells (‘L’) in a tissue-culture dish with the same carbon/free-energy source (1 unit), but without any further cross dependence or cross regulation. The single glucose input flux equally accessible to both cell types was set to 1. For 1 unit of biomass yield, the glucose consumption for heart cells was taken to equal 1.001, and 0.999 for lung cells (these stoichiometries are indicated as four-digit numbers below the beginning of the line of the corresponding process; all other stoichiometries were taken to equal 1 and were therefore not indicated). Flux magnitudes (shown as 3-digit numbers) were obtained by standard FBA of this competitive two-cell-types system, with total biomass production as objective. EX_glucose_e: glucose supply flux to both cells (fixed at 1; negative because it is import); GlcT: glucose transport flux from the outside world to the container with the cells; BiomassH: biomass synthesis flux of the heart cells; GlcTBP: glucose to by-product flux; BiomassL: biomass synthesis flux of lung cells; Byprode: degradation flux for by-product formed.

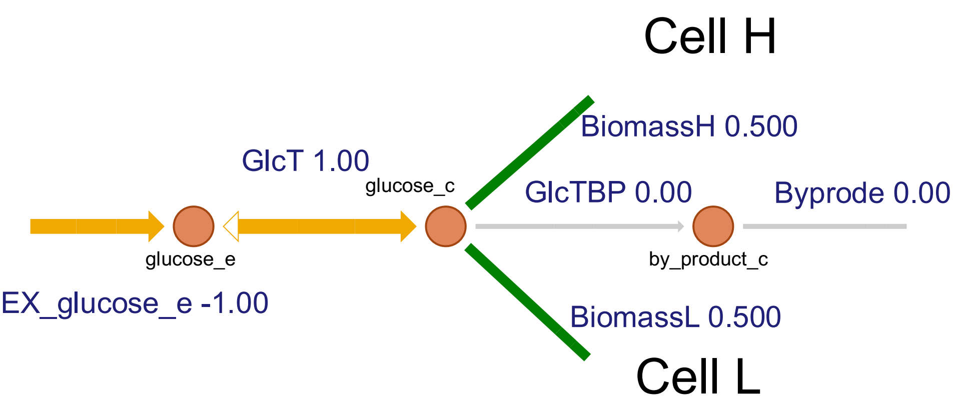

Supplementary Figure 2. One of multiple flux balances predicted for equally expensive cell types 1 and 2 competing for a single carbon/energy source without cross talk. Flux balances

were obtained by standard FBA with total biomass production as objective. The single glucose input flux equally accessible to both cell types was fixed at 1. For 1 unit of biomass yield, the glucose consumption by heart cells and lung cells was taken to be the same. All stoichiometries were herewith taken to equal 1 and were therefore not indicated. Many other possible flux balances were found for these parameter settings and objective function (see Supplementary Table 2).

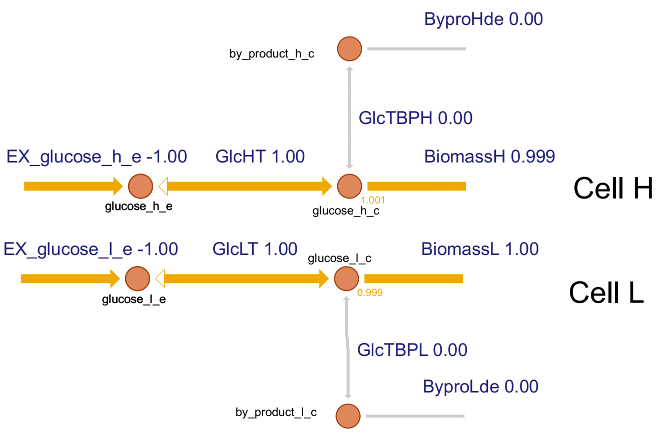

Supplementary Figure 3. Flux balance predicted for cell types 1 and 2 each using their own carbon/free-energy source (*i.e.,* no competition). Only a single flux balance was obtained by standard FBA with total biomass production as objective and without cross talk between the cell types. Both glucose input fluxes were set to 1. An example could be heart cells ('H') and lung cells ('L') each with their own carbon/energy source (1 unit), without any cross dependence and cross regulation. For 1 unit of biomass yield, the glucose consumption by heart cells was taken to equal 1.001; 0.999 for lung cells. EX_glucose_h_e: glucose supply flux to the heart cells fixed at -1; GlcHT: glucose transport flux into the heart cells; BiomassH: biomass synthesis flux for the heart cells; GlcTBPH: glucose to by-product flux for the heart cells; ByproHde: degradation flux of the by-product formed by the heart cells; EX_glucose_l_e: glucose flux fixed at -1 and supplied to the lung cells; GlcLT: glucose transport flux into the lung cells; BiomassL: biomass synthesis flux of lung cells; GlcTBPL: glucose to by-product formation flux by the lung cells; ByproLde: degradation flux for by-product formed by the lung cells.

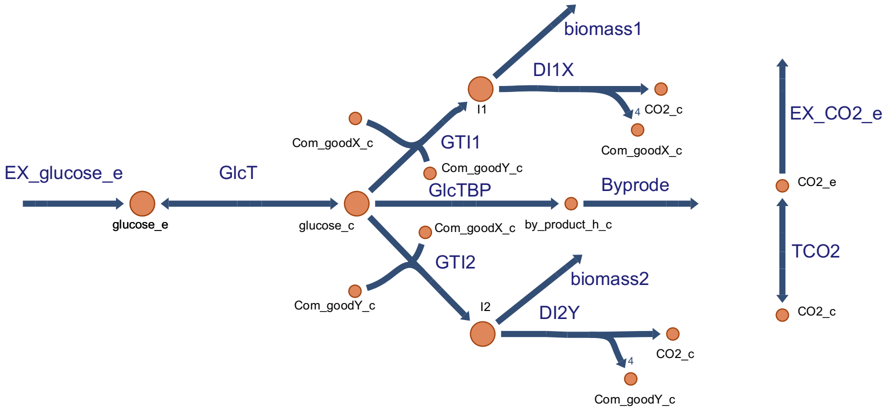

Supplementary Figure 4. The metabolic network used for modeling two cell-types that engaged in metabolic competition for the carbon/free-energy source (glucose) and depended

on each other through the production, one each, of ‘common goods’ (X and Y) that were both essential for their growth. ‘biomass1’ and ‘biomass2’ refer to the biomass synthesis fluxes of cell types 1 and 2, respectively. The glucose flows either to a metabolic intermediate in cell type 1 (*i.e.,* I1) or to such a metabolite I2 in cell type 2. I1 is anabolized into biomass1, or catabolized to X plus CO_2, producing X at a stoichiometry of 4 (stoichiometries different from 1 are indicated next to the arrows.). Similarly, I2 is decomposed into biomass2 or Y, the latter again at the stoichiometry of 4. The reaction from glucose to I1 consumes 1 X and 1 Y per C-mole of glucose and delivers 1 C-mole of I1. The same applies to the reaction from glucose to I2. Both the glucose and the CO_2 exchange reactions are irreversible (inward and outward, respectively, both indicated by _e), and accompanied by reversible transport reactions into/out of the space around the cells.

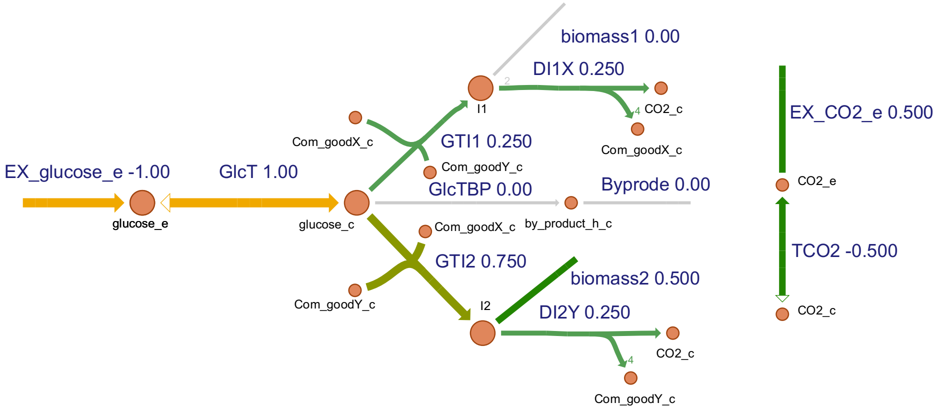

Supplementary Figure 5. Flux balance predicted for cell type 2 and the more ‘expensive’ cell type 1 competing for a single carbon/energy source, and cross dependent on each other through common metabolic goods X and Y. The objective function used by the standard FBA was ‘biomass1+biomass2’. Here, one unit of biomass 1 consumes 2 units of intermediate metabolite I1 (as indicated below the lines from I1 to biomass 1), and one unit of biomass 2 costs one unit of I2 (stoichiometries of 1 are not shown). biomass1 represents the synthesis rate of the ‘expensive’ biomass. Glucose influx was set to 1.

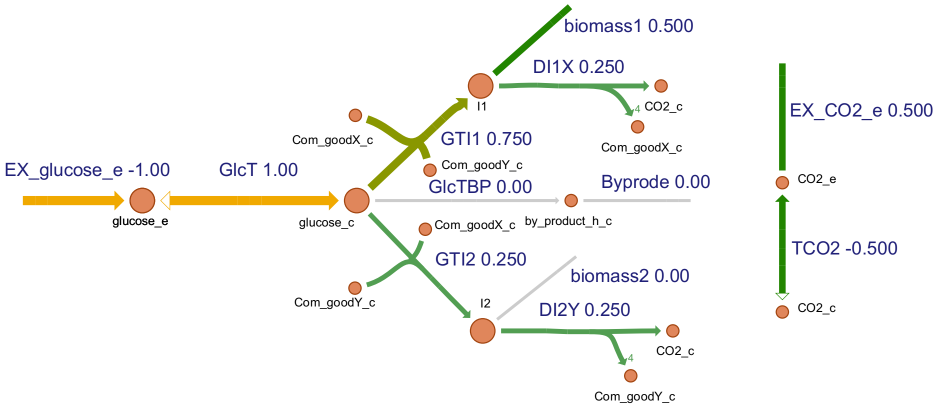

Supplementary Figure 6. Flux balance predicted for cell types 1 and 2 competing for a single carbon/free-energy source, and cross dependent on each other through common metabolic goods (X and Y). One of multiple cases of flux balance predicted for the objective

function ‘biomass1+biomass2’ by standard FBA. The cell types 1 and 2 were taken to have the same biomass synthesis ability (yield per glucose). Glucose influx was fixed at 1.

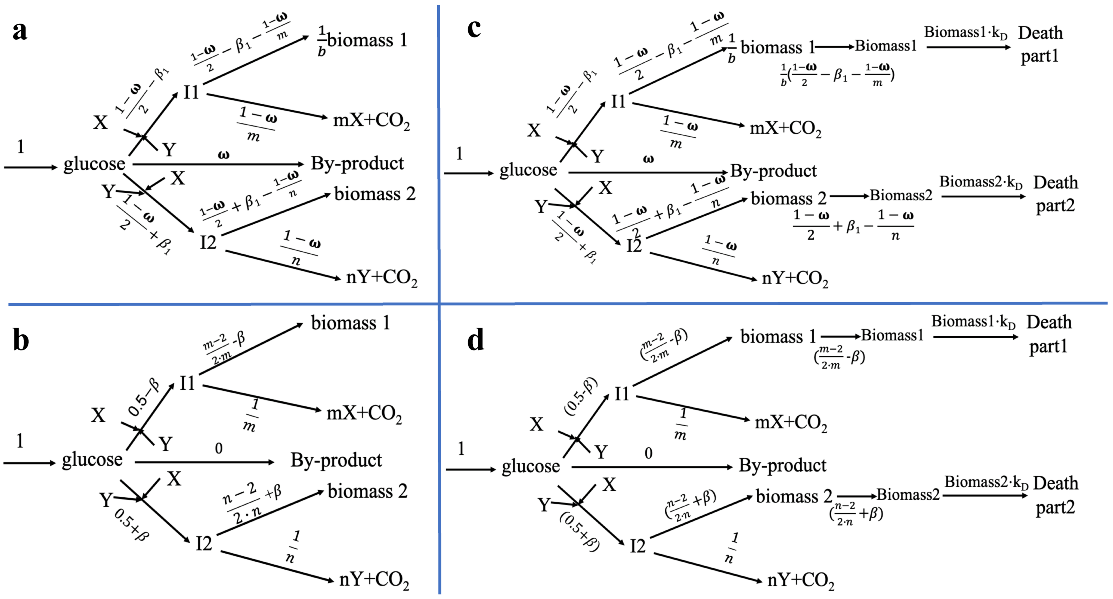

Supplementary Figure 7. Network structure of and optimal balanced fluxes for two cell types competing for a common substrate, depending on common goods X and Y, and growing (c, d). Stoichiometries equal 1 unless indicated by a small digit in front of the reactant (biomass 1: 1/b; X and Y: m and n, respectively). Balanced flux values for the optimal state of maximum total biomass production (in C-mol/time unit) are written above or below the reaction arrows. a: The *metabolic* network in which CO_2 and biomass of both cell types constitute the output, whilst glucose, I1, I2, X and Y are balanced metabolites. b: The optimal balanced flux pattern (FBA solution) through the network of a with b=1. c: The growth and metabolic network with additional balances around the two biomass concentrations. d: Balanced fluxes for the optimal state, defined by the total biomass production flux being maximal, as obtained whenever biomass 1 is equally expensive as biomass 2 (*i.e.,* b=1). For b=1, the glucose consumption bias is equal to biomass synthesis bias (i.e., $\beta=\beta_1$).

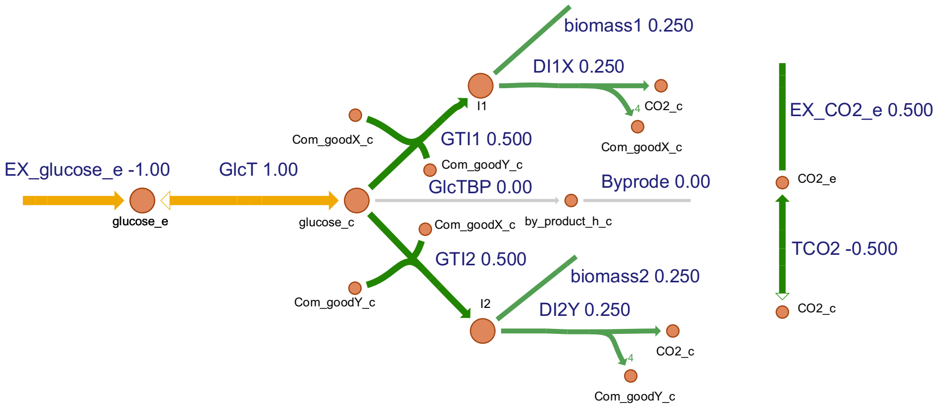

Supplementary Figure 8. Flux balance predicted for cell types 1 and 2 competing for a single carbon/free-energy source, and cross dependent on each other through common

metabolic goods (Objective function=biomass1+biomass2). Cell type 1 and cell type 2 were taken to have the *same* specific growth rate of 0.25/*ts*. Glucose influx was fixed at 1/*ts*.

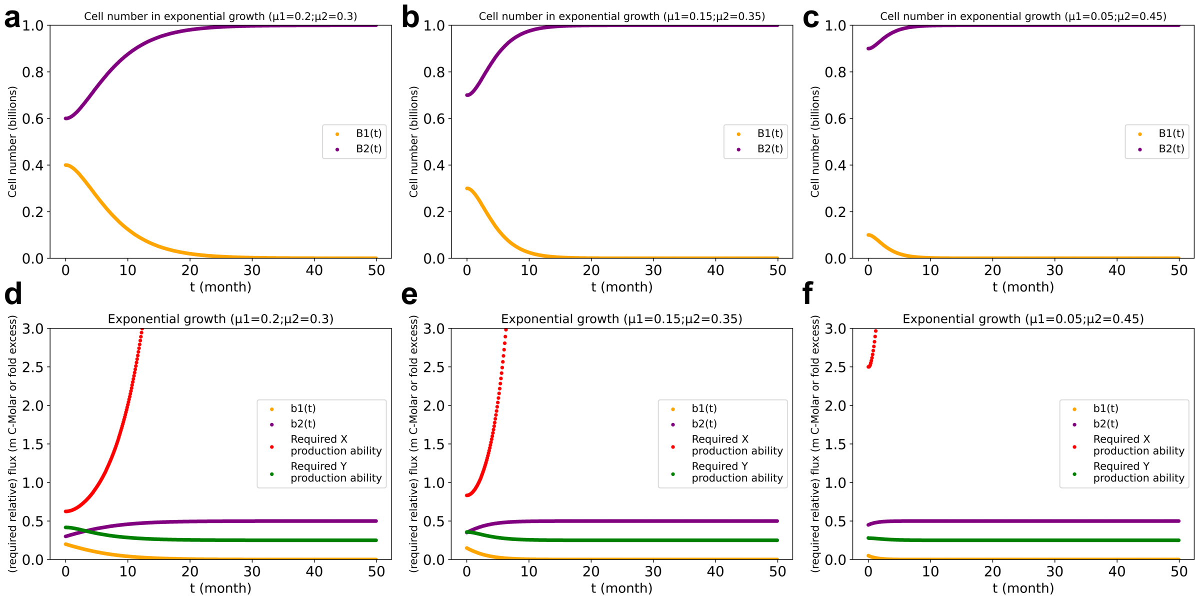

Supplementary Figure 9. Cell number changed in time calculated for the *exponential* growth FBA method for the three β values indicated and their related biomass synthesis rate and required X and Y production ability in *unlimited capacities* situation. The equations discussed in Materials and Methods were integrated over time k_D=0.5 and *ts*=0.1, for *β*=0.05 (a and d), 0.1 (b and e), or 0.2 (c and f). $\mu_1 = 0.25 - \beta$; $\mu_2 = 0.25 + \beta$. The orange points and purple points (picture a, b and c) represent the cell numbers of cell type 1 (B1) and of cell type 2 (B2), respectively. The orange points and purple points (picture d, e and f) represent the biomass synthesis rate of cell type 1 and of cell type 2, respectively. The red points and green points (picture d, e and f) represent the required X and Y production ability of cell type 1 and of cell type 2, respectively. B1(0)=0.4 and B2(0)=0.6 for β=0.05; B1(0)=0.3 and B2(0)=0.7 for β=0.1; B1(0)=0.1 and B2(0)=0.9 for β=0.2 (These values were the same for subsequent calculations unless specifically stated).

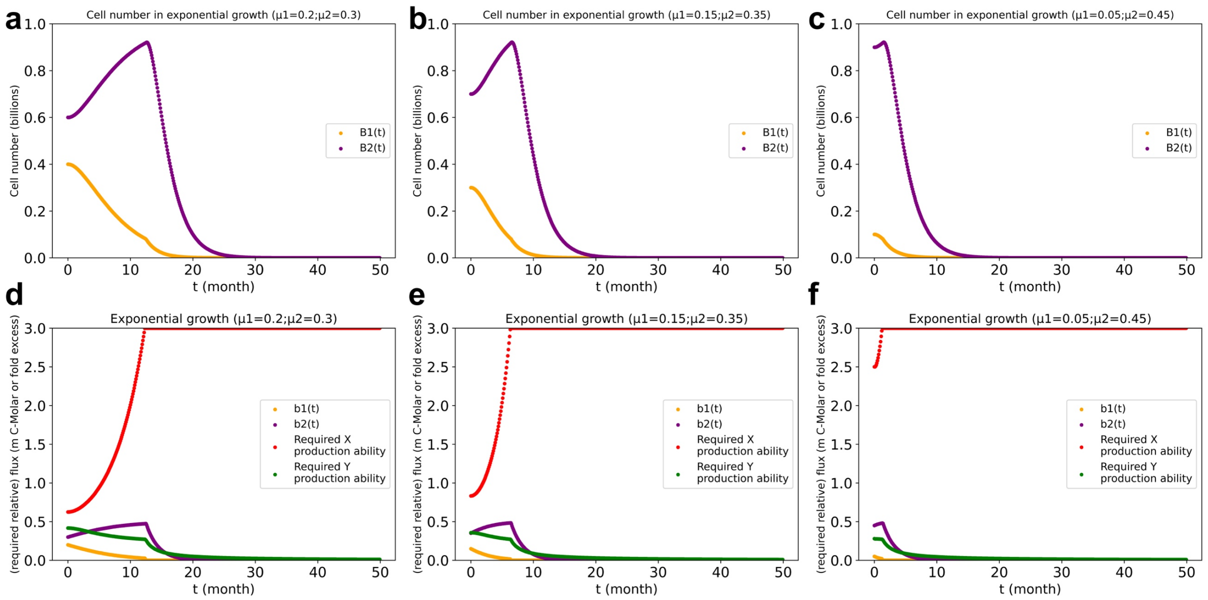

Supplementary Figure 10. Biomass levels (B1, B2) and related biomass synthesis rates (b1 and b2) and the required X and Y production rates for cell type 1 and cell type 2, calculated for different specific growth bias values (0.05 (a and d), 0.1 (b and e), 0.2 (c and f)) and with capacities of metabolic reactions limited through '*exponential*' competition as described under Supplementary Materials. Glucose influx was unlimited and glucose efflux absent. The FBA objective was maximal total biomass synthesis. These were calculated by adding the balance between b1 and b2 and the death rate (Biomass 1 or Biomass 2 respectively, multiplied by k_D=0.5/*ts*) after every time step of 0.1 month. A total specific biomass synthesis of 0.5 requires an X synthesis flux of 0.25. The maximum synthesis flux of X catalyzed by cell type 1 was taken to equal 3 per unit Biomass 1. Hence at a cell number of 0.25/3=0.083 the amount of B1 stopped to suffice for the synthesis of the X required for the maximum total biomass synthesis of 0.5, leading to the drops in the optimal b1 and b2 predicted by the FBA. It was checked that the FBA solutions obtained corresponded to flux balances throughout the metabolic network.

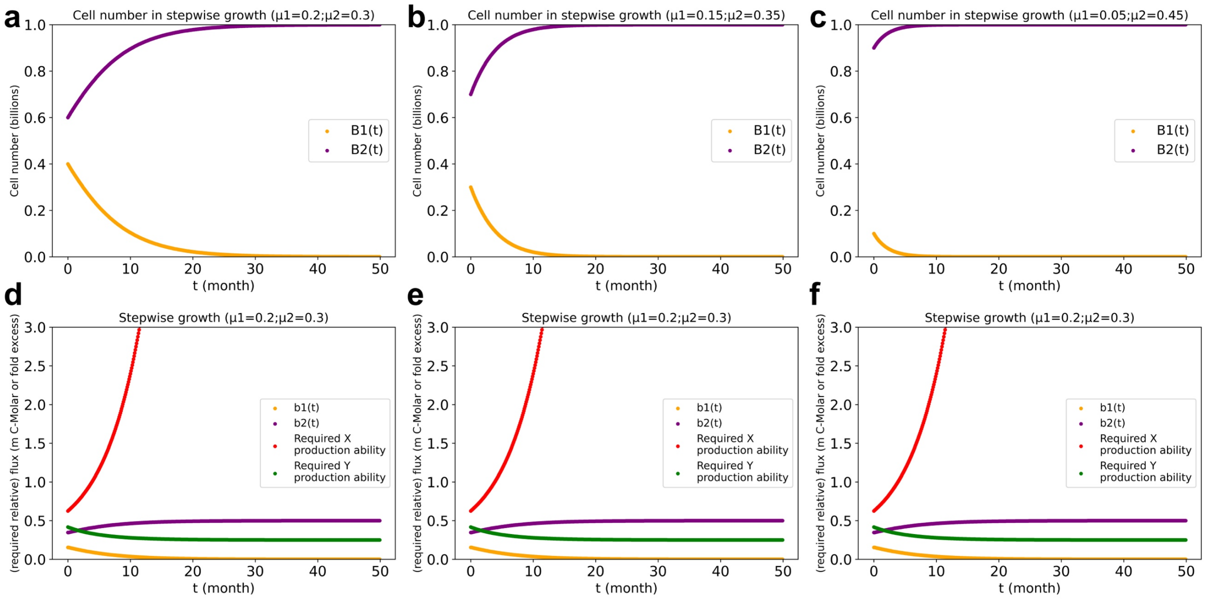

Supplementary Figure 11. Calculated cell number changing with time by dFBA for *stepwise growth* with three β values (0.05(a and d), 0.1(b and e), 0.2(c and f)) and their

related biomass synthesis rate and required X and Y production ability in *unlimited capacities* situation. The equations discussed in Materials and Methods were used for calculation ($\mu_1 = 0.25 - \beta$; $\mu_2 = 0.25 + \beta$). The orange points and purple points (picture a, b and c) represent the cell numbers of cell type 1 (B1) and of cell type 2 (B2), respectively. The orange points and purple points (picture d, e and f) represent the biomass synthesis rate of cell type 1 and of cell type 2, respectively. The red points and green points (picture d, e and f) represent the required X and Y production ability of cell type 1 and of cell type 2, respectively.

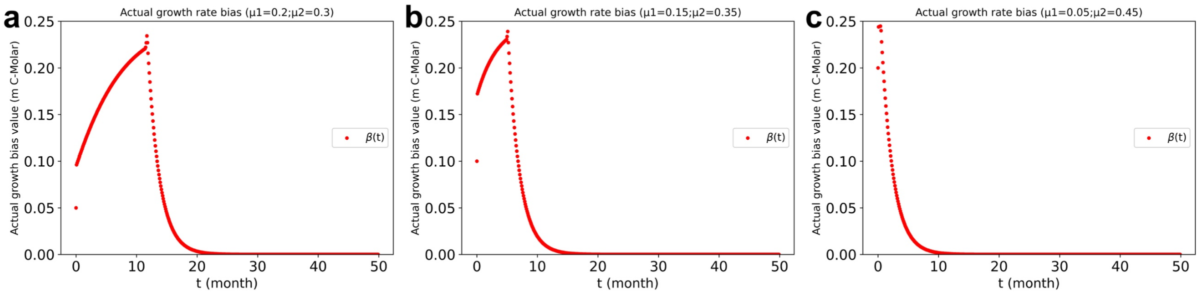

Supplementary Figure 12. Actual growth bias ($\beta(t) \stackrel{\text{def}}{=} (b_2(t) - b_1(t))/2$) in stepwise growth at various original growth rate biases β (0.05(a), 0.1(b), 0.2(c)).

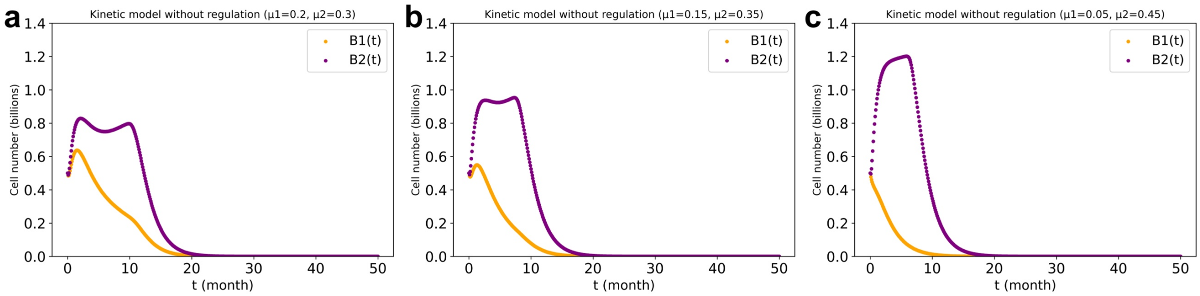

Supplementary Figure 13. Calculated cell number changing with time for different β values (0.05(a), 0.1(b), 0.2(c)) in the kinetic model by using Copasi. The initial values were: $B_1(0)=B_2(0)=0.5$; $Glc(0)=I_1(0)=I_2(0)=X(0)=Y(0)=1$. The rate constants for each equation are according to the different growth biases, as shown in Supplementary Table 6.

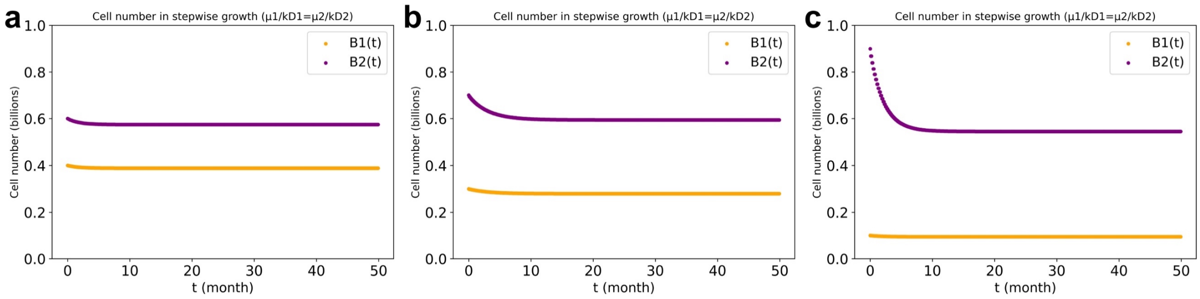

Supplementary Figure 14. Calculated cell number changing with time by dFBA for stepwise growth with different β values (0.05(a), 0.1(b), 0.2(c)). Here the relationship for growth rate and death rate for two cell types satisfies this equation '$\frac{\mu_1}{k_{D1}} = \frac{\mu_2}{k_{D2}}$'. In a: $\mu_1 = 0.2, k_{D1} = 0.4, \mu_2 = 0.3, k_{D2} = 0.6$; In b: $\mu_1 = 0.15, k_{D1} = 0.3, \mu_2 = 0.35, k_{D2} = 0.7$; In c: $\mu_1 = 0.05, k_{D1} = 0.1, \mu_2 = 0.45, k_{D2} = 0.9$.

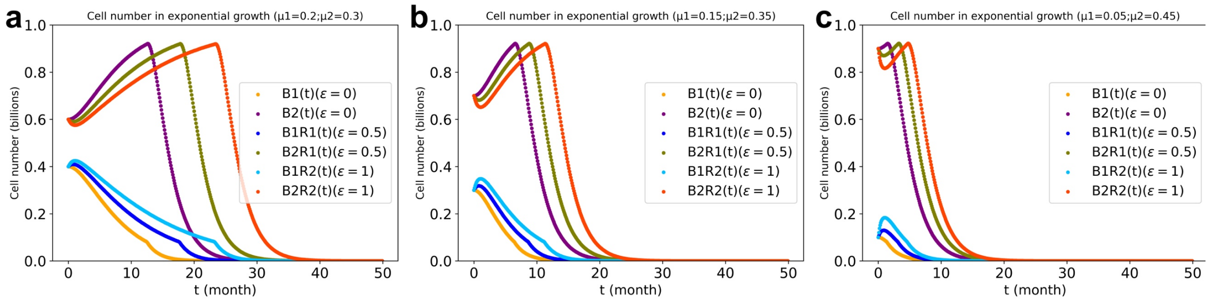

Supplementary Figure 15. Exponential growth of two cell types' system at various regulation powers ε and growth rate biases β. $\beta \stackrel{\text{def}}{=} (\mu_2 - \mu_1)/2$=0.05(a), 0.1(b), 0.2(c), and with capacities of metabolic reactions limited as in Supplementary Fig. 10. The value of ε indicates the strength of the regulation between the two cell types. The orange points show the cell number of cell type 1, the purple points show the cell number of cell type 2 (both are without regulation). The blue points and olive points are with regulation power of 2, for cell type 1 and cell type 2, respectively. The deep-sky-blue points and orange-red points are with regulation power of 5, for cell type 1 and cell type 2, respectively. The preset-exponential-growth-with-regulation algorithm was used for these dcFBA computations with total biomass synthesis as objective function.

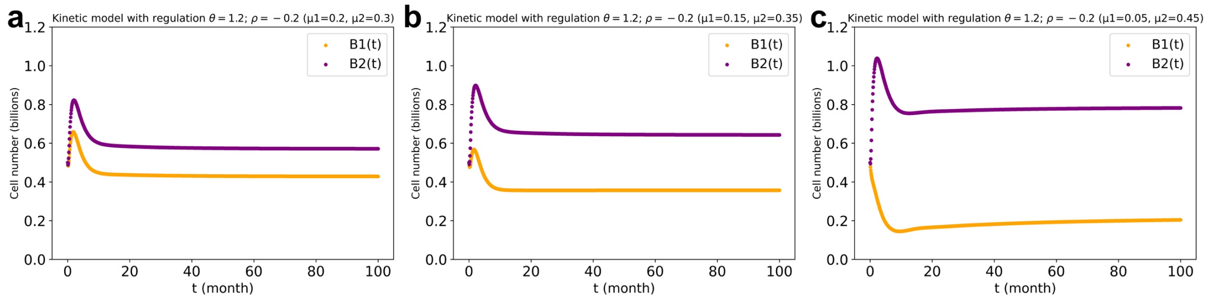

Supplementary Figure 16. The regulation produced by the kinetic model for growth bias of 0.05(a), 0.1(b), 0.2(c). The regulation power θ (regulation from glucose to each inter-metabolites) was taken to equal 1.2, the regulation power ρ for common goods production was taken to equal -0.2 and with product inhibition. For the kinetic details see Supplementary materials.

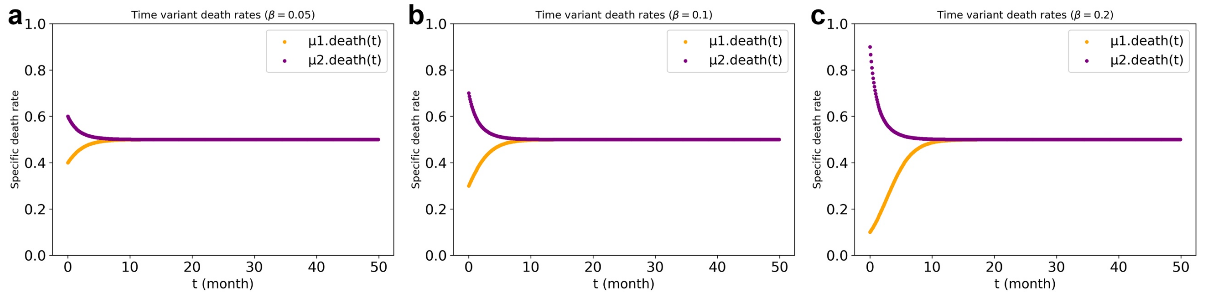

Supplementary Figure 17. Time variant death rates in three growth biases with constant biomass synthesis rate. (a) $\beta = 0.05$; (b) $\beta = 0.1$; (c) $\beta = 0.2$. For each point of death rate, it satisfies the biomass synthesis rate equals to the death part.

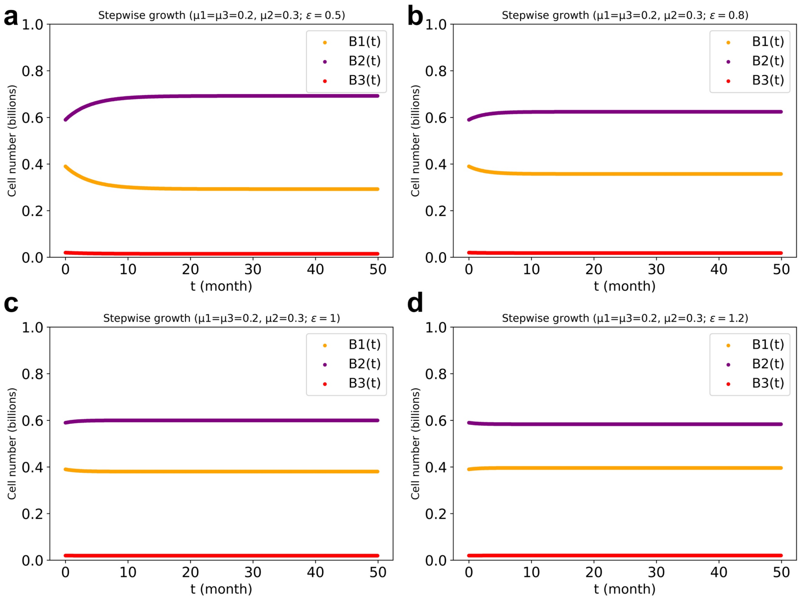

Supplementary Figure 18. Cell numbers in the three-cell types system with various regulation powers (ε) between cell type 1 and type 2, and also between cell type 3 and type 2. The third cell type had same specific growth rate as cell type 1 (0.2 ts^{-1}). (B1, B2 and B3 are the biomass values (cell numbers) for cell type 1, cell type 2 and cell type 3, respectively). The stepwise growth algorithm in three-cell types system with cross-regulation between cell type 1 and type 2, and also cell type 3 and type 2 (i.e., 'the control case') was used for the dcFBA computations.

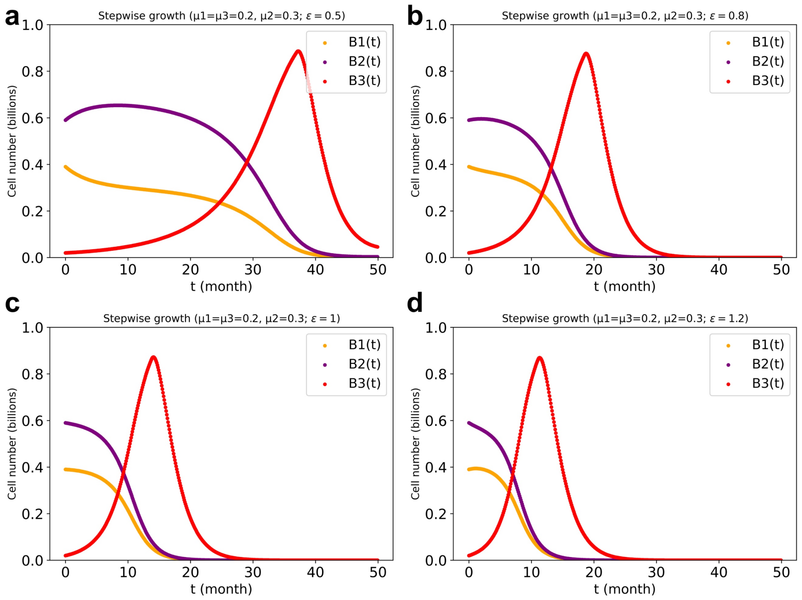

Supplementary Figure 19. Cell numbers in the three-cell types system with various regulation powers (ε) only between cell type 1 and type 2. The third cell types had same specific growth rate as cell type 1 (0.2 ts^{-1}). (B1, B2 and B3 are the biomass values (cell numbers) for cell type 1, cell type 2 and cell type 3, respectively). The stepwise growth algorithm in three-cell types system with only cross-regulation between cell type 1 and type 2 (i.e., 'the non-responsive and non-communicating case 1') was used for the dcFBA computations.

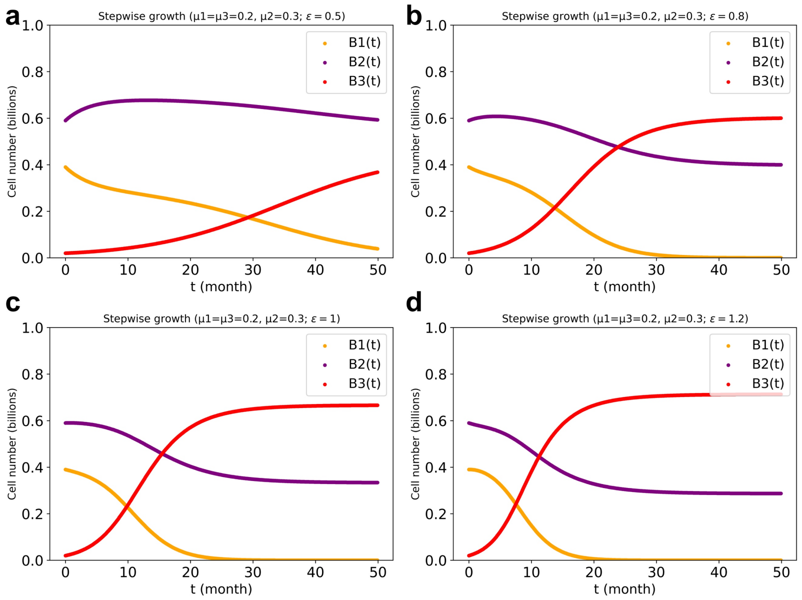

Supplementary Figure 20. Cell numbers in the three-cell types system with various regulation powers (ε) between cell type 1 and type 2, and from cell type 3 to type 2, but without regulation of cell type 3 by cell type 2 ('The non-responsive Case 2'). The third cell type had the same specific growth rate as cell type 1 (0.2 ts^{-1}). (B1, B2 and B3 are the biomass values (cell numbers) for cell type 1, cell type 2 and cell type 3, respectively). The stepwise growth algorithm in three-cell types system of the non-responsive case 2 was used for the dcFBA computations.

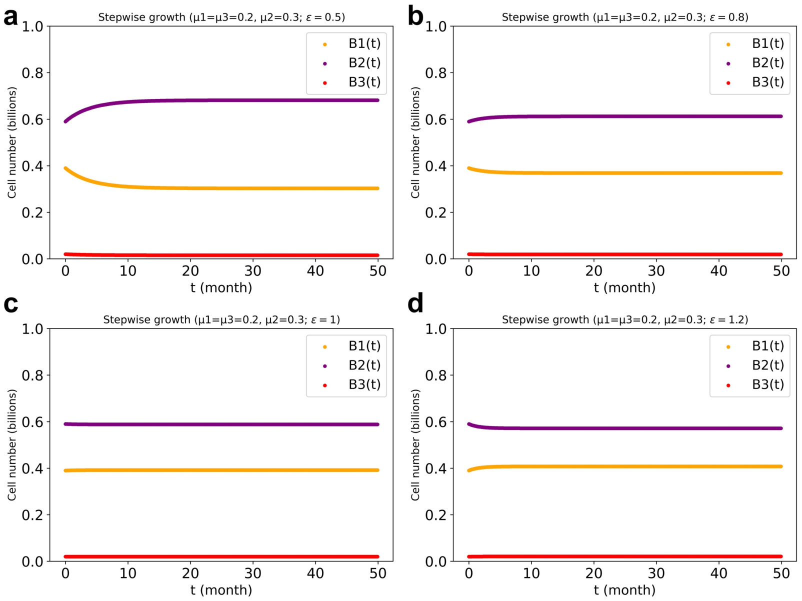

Supplementary Figure 21. Cell numbers in the three-cell types system with various regulation powers (ε) between cell type 1 and type 2, and from cell type 2 to type 3, but without regulation of cell type 2 by cell type 3 ('The non-communicating case 3'). The third cell types had same specific growth rate as cell type 1 (0.2 ts^{-1}). (B1, B2 and B3 are the biomass values (cell numbers) for cell type 1, cell type 2 and cell type 3, respectively). The stepwise growth algorithm in three-cell types system of the non-communicating case 3 was used for the dcFBA computations.

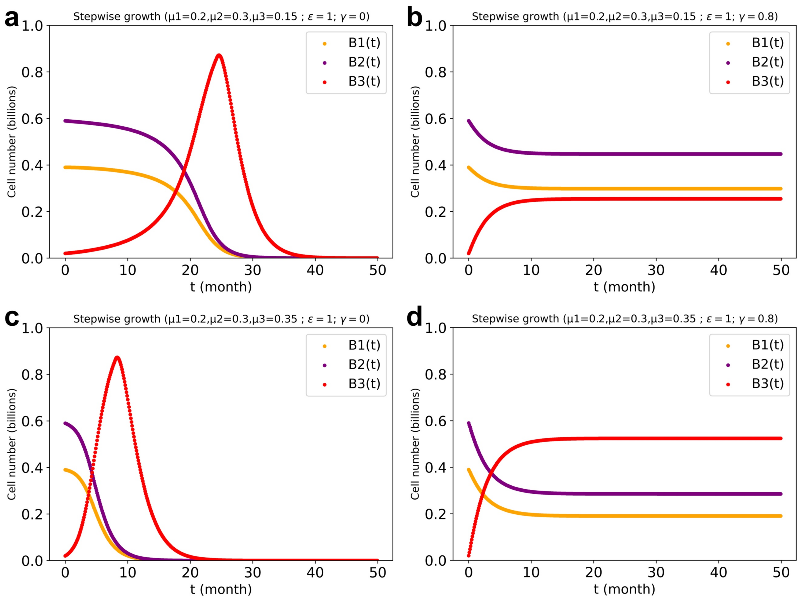

Supplementary Figure 22. Cell number developing over with time in the three cell types system: with lower inherent specific growth rates for cell type 3 (μ_3=0.15< μ_1=0.2< μ_2=0.3/*ts* (a and b)); with higher inherent specific growth rates for cell type 3 (μ_1=0.2< μ_2=0.3< μ_3=0.35/*ts* (c and d)). a and c represent no regulation among the cell type 3 and other two cell types; b and d represent there exists regulation power of 0.8 between cell type 3 and other two cell types. The stepwise growth FBA algorithm in the three cell types' system with a cross-regulation power of 1 between cell type 1 and type 2 and reciprocal regulation at strength γ between cell type 3 and the other two cell types was used for the dcFBA computations.

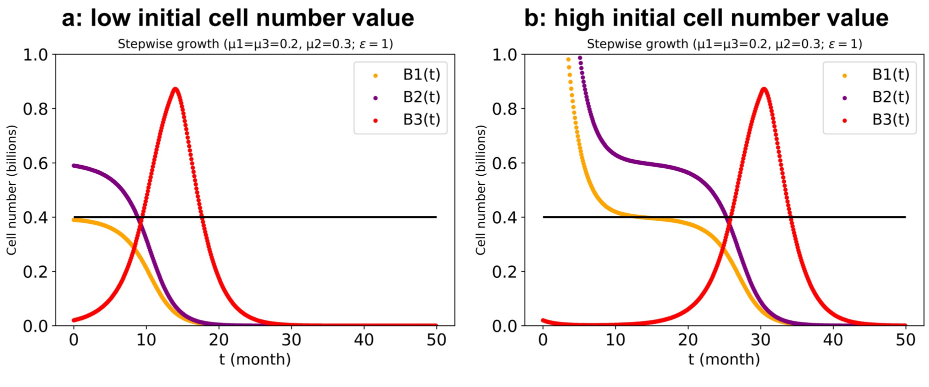

Supplementary Figure 23. Cell number values ('B1', 'B2', 'B3') in the three-cell types system with regulation power (ε) of 1 between cell type 1 and type 2, and no cross regulation among three cell types (γ=0). (a) represents the low initial cell number (i.e., $B_1(0)$=0.39, $B_2(0)$=0.59, $B_3(0)$=0.02); (b) represents a high initial cell number (i.e., $B_1(0)$=3.9, $B_2(0)$=5.9, $B_3(0)$=0.02). The third cell types had same specific growth rate as cell type 1 (0.2 ts^{-1}). We set the cell number value of 0.4 billion for cell type 3 ('B3') as critical value for the system, shown by the solid horizontal black line. When the transformed cell (type 3) number exceeds 0.4 billion, the system will become unstable. The stepwise growth algorithm in three-cell types system with cross-regulation power of 1 between cell type 1 and type 2 was used for the dcFBA computations.

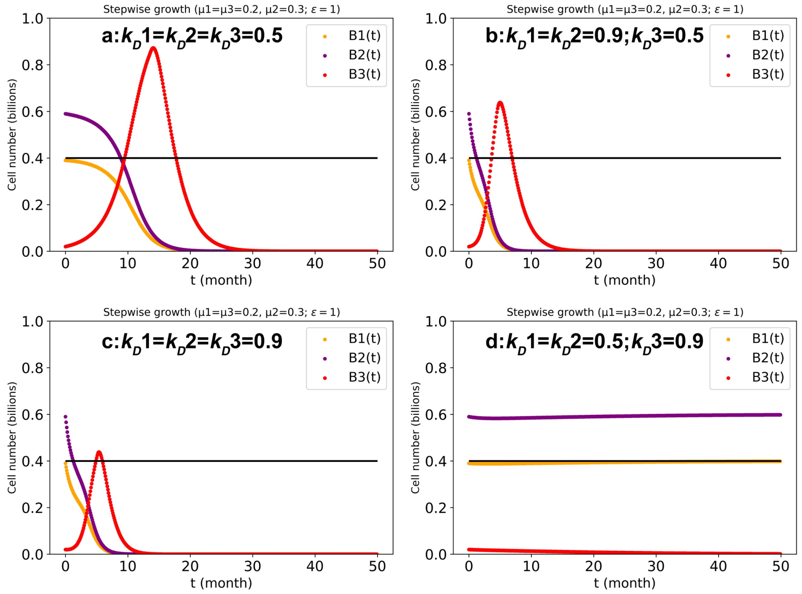

Supplementary Figure 24. Comparing the effect of death rate (k_D) on cell number in the three cell types system. The cell number of 0.4 billion is again the critical value for the transformed cells. 'k_D1', 'k_D2' and 'k_D3' are the death rate for cell type 1, type 2 and type 3, respectively. (a) represents the same death rate (i.e., 0.5/*ts*) for three cell types; (b) represents death rate for normal cells (i.e., 0.9/*ts* to 0.5/*ts* for type3); (c) represents the increased death rate (i.e., 0.9/*ts*) for all cell types; (d) represents death rate for type 3 (i.e., 0.9/*ts* to 0.5/*ts* for normal cells). The stepwise growth algorithm in three cell types system with cross-regulation power of 1 between cell type 1 and type 2 was used for the dcFBA computations.

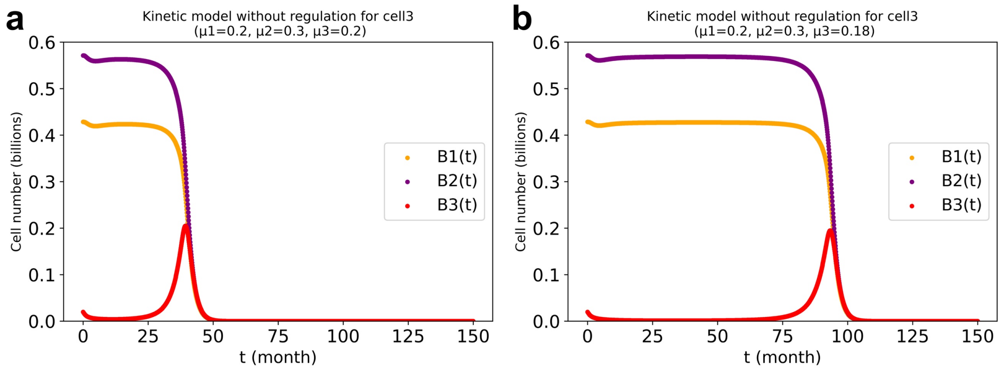

Supplementary Figure 25. Calculated cell number changing with time for same (a) or lower (b) inherent growth rate as cell type 1 for type 3 in the kinetic model by using Copasi. For the kinetic details see Supplementary materials.

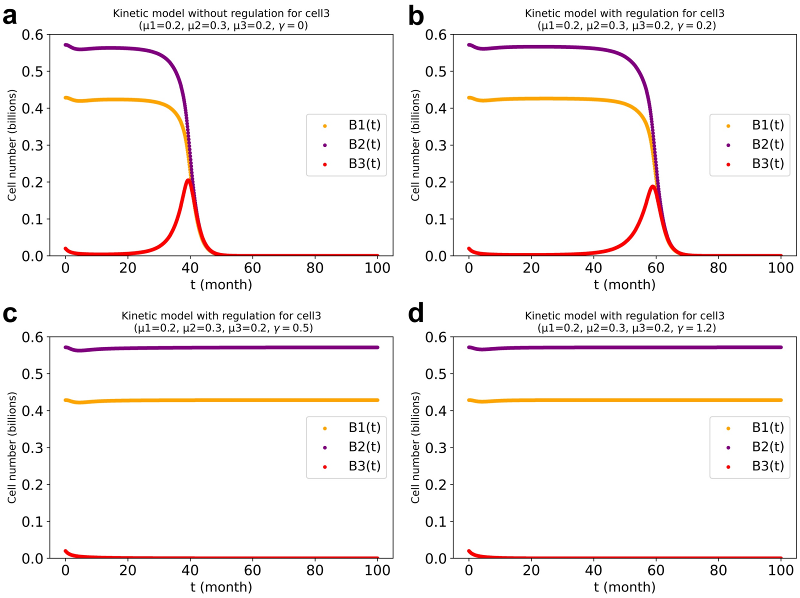

Supplementary Figure 26. The regulation produced by the kinetic model for cell type 3 with growth rate of $\mu_1 = 0.2/ts$; $\mu_2 = 0.3/ts$; $\mu_3 = 0.2/ts$. The regulation power γ (regulation from glucose to I3) was taken to 0 (a), 0.2 (b), 0.5 (c) and 1.2 (d). For the kinetic details see Supplementary materials.

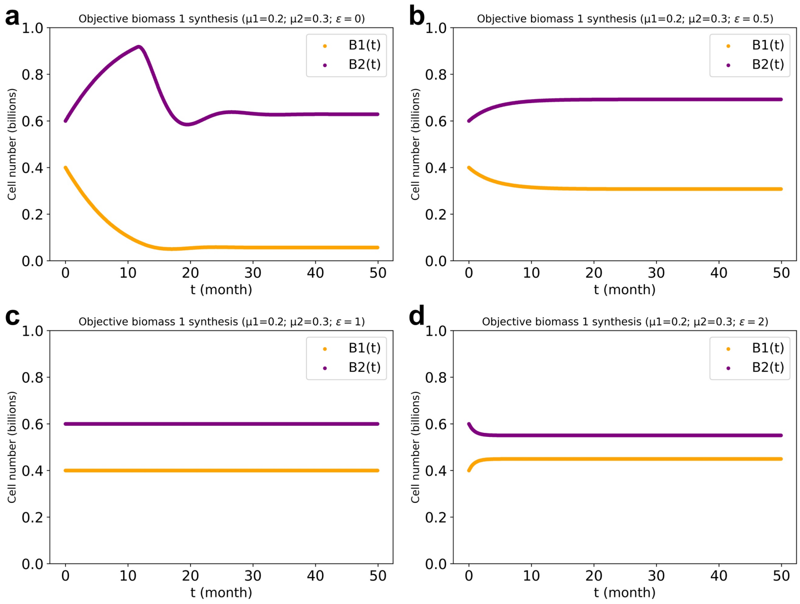

Supplementary Figure 27. The two cell types system with biomass 1 production as objective function at various magnitudes of the regulation power ε with growth rate bias of 0.05, and with capacities of metabolic reactions limited as in Fig. 2. a: $\varepsilon = 0$ (no such cross regulation). b: $\varepsilon = 0.5$. c: $\varepsilon = 1$. d: $\varepsilon = 2$. The stepwise-growth-with-regulation FBA algorithm was used.

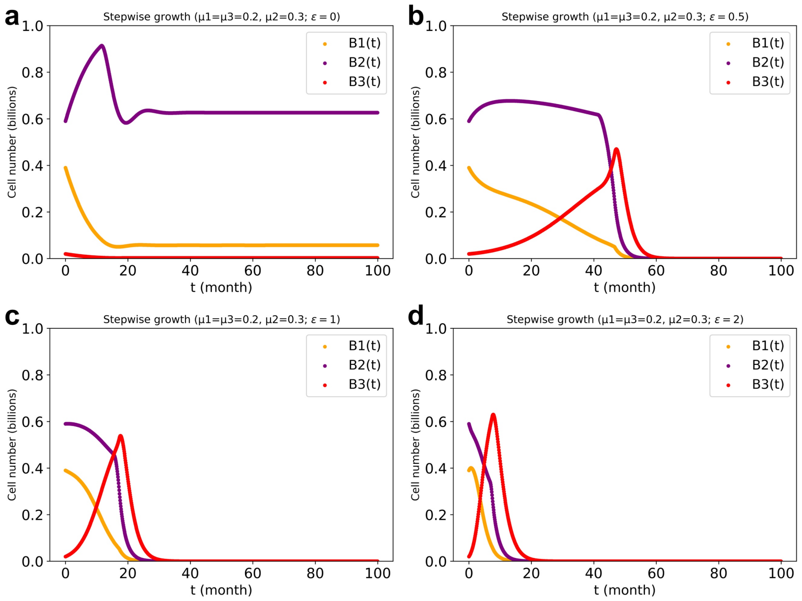

Supplementary Figure 28. Cell numbers in the three-cell types system with ‘biomass 1+ biomass 3’ production as objective function at various regulation powers (ε), but only between cell type 1 and type 2. The third cell types had the same specific growth rate as cell type 1 (0.2 ts^{-1}). B1, B2 and B3 are the biomass values (cell numbers) for cell type 1, cell type 2 and cell type 3, respectively. The stepwise growth algorithm in three-cell types system with only cross-regulation between cell type 1 and type 2 (i.e., ‘the non-responsive and non-communicating case 1’) was used for the dcFBA computations.

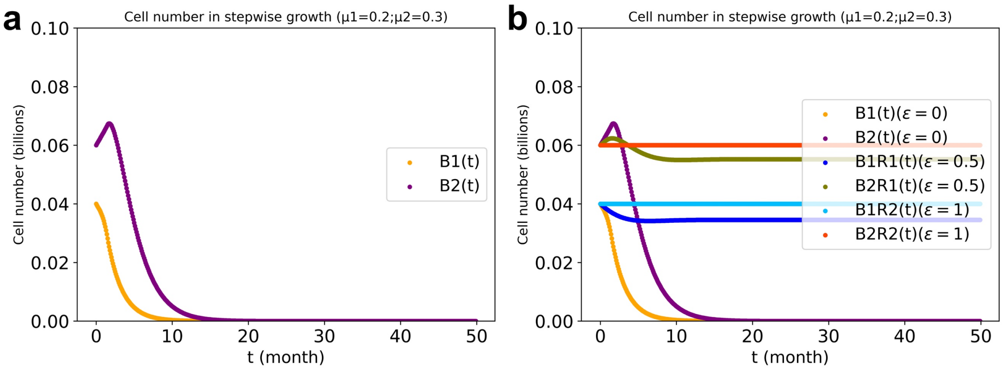

Supplementary Figure 29. Cell numbers in the two-cell types system with total biomass production as objective function, calculated for specific growth bias value of 0.05. The stepwise growth algorithm in two-cell types system without (figure a) and with (figure b) cross-regulation between cell type 1 and type 2 was used for the dcFBA computations.

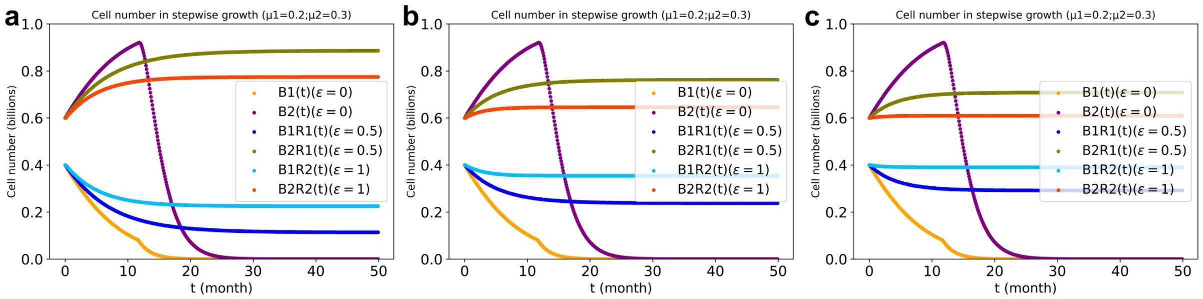

Supplementary Figure 30. Cell numbers in the two-cell types system with total biomass production as objective function, calculated for specific growth bias value of 0.05. All three cases are with regulation. a: $\frac{K_d}{\alpha}$=0.2; b: $\frac{K_d}{\alpha}$=1; c: $\frac{K_d}{\alpha}$=5.The stepwise growth algorithm in two-cell types system with cross-regulation between cell type 1 and type 2 was used for the dcFBA computations.

Supplementary Tables

Reaction ID	Minimum flux	Maximum flux
**EX_glucose_e (fixed)**	-1.000	-1.000
**BiomassH**	0.000	0.000
**BiomassL**	1.001	1.001
**Byprode**	0.000	0.000

Supplementary Table 1. Possible flux ranges in the substrate competition network with different glucose costs of the two cell types' biomass synthesis (Supplementary Fig. 1). For each process the flux range that can still produce the maximum objective value (by the other fluxes adjusting) is shown, as obtained by FVA. (For 1 unit of biomass yield, the glucose consumption per unit biomass synthesized was taken as 1.001 for heart cells (H) and 0.999 for lung (L) cells. Glucose influx was fixed at 1. Flux units are C-moles/arbitrary time unit).

Reaction ID	Minimum flux	Maximum flux
**EX_glucose_e**	-1.0	-1.0
**BiomassH**	0.0	1.0
**BiomassL**	0.0	1.0
**Byprode**	0.0	0.0

Supplementary Table 2. Possible flux ranges in the substrate competition network in which the two biomasses are equally expensive (Supplementary Fig. 2), as obtained by FVA. For each process the flux range is shown that can still produce the maximum objective value (by the other fluxes adjusting), as obtained by FVA. The two cell types were assumed to have the same growth yield on glucose. Flux units are C-moles/arbitrary time unit.

Reaction ID	Minimum flux	Maximum flux
**EX_glucose_e**	-1.00	-1.00
**GTI1**	0.25	0.25
**GTI2**	0.75	0.75
**biomass1**	0.00	0.00
**biomass2**	0.50	0.50
**DI1X**	0.25	0.25
**DI2Y**	0.25	0.25
**EX_CO2_e**	0.50	0.50

Supplementary Table 3. Possible flux ranges for two cell types competing for substrate and interacting through common goods where the two cell types differed in terms of glucose expense per unit biomass (the network of Supplementary Fig. 5). For each process the flux range is shown that is still compatible with the same maximum total growth rate value (by the other fluxes adjusting, as obtained by FVA). The two biomasses were taken to be differently expensive. Flux units are C-moles/arbitrary time unit.

Reaction ID	Minimum flux	Maximum flux
**EX_glucose_e**	-1.00	-1.00
**GTI1**	0.25	0.75
**GTI2**	0.25	0.75
**biomass1**	0.00	0.50
**biomass2**	0.00	0.50
**DI1X**	0.25	0.25
**DI2Y**	0.25	0.25
**EX_CO2_e**	0.50	0.50

Supplementary Table 4. Possible flux ranges for two equally expensive cell types competing for substrate and interacting through common goods (the network of Supplementary Fig. 6 and Supplementary Fig. 8). For each process the flux range is shown that is still compatible with the same maximum total growth rate value (by the other fluxes adjusting, as obtained by FVA). The two biomasses were taken to be equally expensive. Flux units are C-moles/arbitrary time unit.

Reaction Name	Reaction Equation	Rate law
**Glc to I_1**	Glc + Y + X -> I_1; B_1	v=k_{R1}·[Glc]·[X]·[Y]·[B_1]
**I_1 to b_1**	I_1->b_1; B_1	v=k_{R2}·[I_1]·[B_1]
**I_1 to X**	I_1->4·X + CO_2; B_1	v=k_{R3}·[I_1]·[B_1]
**Glc to by-product**	Glc->by-product;	v=k_{R4}·[Glc]
**Glc to I_2**	Glc + X + Y -> I_2; B_2	v=k_{R5}·[Glc]·[X]·[Y]·[B_2]
**I_2 to b_2**	I_2->b_2; B_2	v=k_{R6}·[I_2]·[B_2]
**I_2 to Y**	I_2->4·Y + CO_2; B_2	v=k_{R7}·[I_2]·[B_2]
**b_1 to B_1**	b_1->B_1;	v=k_{R8}·[b_1]
**b_2 to B_2**	b_2->B_2;	v= k_{R9}·[b_2]
**Glc out to Glc in**	-> Glc	v=k_{R10}
**Death of Cell type 1**	B_1 ->	v=k_{R11}·[B_1]
**Death of Cell type 2**	B_2 ->	v=k_{R12}·[B_2]

Supplementary Table 5. Reaction and rate equations of the kinetic model. The values of the rate constant are shown in Supplementary Table 6.

Rate Constant	β=0.05	β=0.1	β=0.2
k_{R1}	9	8	6
k_{R2}	4	3	1
k_{R3}	5	5	5
k_{R4}	0.001	0.001	0.001
k_{R5}	11	12	14
k_{R6}	6	7	9
k_{R7}	5	5	5
k_{R8}	1	1	1
k_{R9}	1	1	1
k_{R10}	1	1	1
k_{R11}	0.5	0.5	0.5
k_{R12}	0.5	0.5	0.5

Supplementary Table 6. The rate constant values for all reactions with three growth biases in two cell types system.

Supplementary notes

1. FBA of the metabolic network for cells just competing for common substrate---No co-existence

Multicellular organisms usually have a single food uptake and circulation system for all their cells: the cells will have to share in or compete for a single supply of food. This suggests that a cell type with a higher inherent specific growth rate will not only grow faster than the others, but also deplete the common substrate, cause the other cell types to stop growing, and thereby obliterate stable co-existence. We here examine whether this argumentation is supported by FBA.

For simplicity we shall focus on two cell types together forming an 'organism' and competing for the same glucose, which is supplied at a constant rate (Supplementary Fig. 1). New biomass that is synthesized is assumed to leave the system and not to participate in further growth. The flux balance requested is around metabolic intermediates, not around the biomass levels. In general, cell types will differ in tasks they carry out. Thereby the amount

of glucose they consume per amount of biomass they produce (*i.e.,* the glucose stoichiometry) may differ between them. FBA is oblivious of kinetic detail, but it does have such substrate consumption and product production stoichiometries on board for every reaction.

Our first question was whether at maximum total growth rate there could be any stable coexistence if two cell types competed for growth substrate. We computed the optimal steady-state flux pattern by standard FBA using total biomass synthesis flux as objective function. The answer was ‘No’ (Supplementary Fig. 1, the thick orange lines). Even for the slightest advantage for the biomass reaction of cell type L, the balance of fluxes flipped *entirely* towards that cell type: no co-existence. Flux variability analysis (FVA) showed that this was the only optimal steady flux pattern for that difference in growth yield between the two cell types (Supplementary Table 1). The sole exception was when the two growth yields were taken equal. Then a stable co-existence would be one (but not the only) steady-state metabolic flux pattern with optimal total biomass production flux (Supplementary Fig. 2 and Supplementary Table 2) if we did not consider the death of cells. Paradoxically, removing the competition for growth substrate led to some sort of stable co-existence in the state of maximal total growth rate even if the two cell types differed in growth yield. Then their biomass synthesis rates differed in proportion to the yield differences, but neither became equal to zero (Supplementary Fig. 3). As we consider it unlikely that the different cell types of a multicellular organism would persistently all have the same growth yield, we conclude that competition for growth substrate is at odds with co-existence of cell types in the optimal state (both at maximum total growth rate) and that the reason for this is the competition. As shown in Supplementary Fig. 3 in which there is no competition for common substrate, both biomasses were synthesized in the optimal states of maximum total biomass synthesis case, even though they differed in glucose-cost.

2. Standard FBA with cell-cell competition and metabolic cross dependence (failing to produce stability)

Competition for common metabolic substrates did not suffice to produce co-existence. To examine what may then cause co-existence, we incorporate the concept of interdependence observed in multi-tissue organisms, where different tissues support each other. For instance, ‘lung cells’ serve ‘heart cells’ (and themselves) by transferring oxygen to the blood (and secreting CO_2 from it) whilst the ‘heart cells’ serve the ‘lung cells’ by pumping blood with glucose and oxygen to both cell types. And, ‘lung cells’ provide oxygen to both ‘liver cells’ and ‘lung cells’, whilst ‘liver cells’ provide glutamine as nitrogen source to both. (We put the cell types between apostrophes because reality is more complex and tissues rather than single cell types are involved.) To test our concept, we added to the model that the cell types need each other metabolically: each cell type produced and secreted a unique metabolite serving as ‘common good’ for both cell types. Supplementary Fig. 4 shows a network diagram where there is a fixed supply of the substrate glucose, which can be used either by cell type 1 (the upper branch in the diagram), or by cell type 2 to make new cells. X and Y are called ‘common goods’ because both are required by each cell type to convert glucose into intermediate I1 or I2. In a multicellular organism, these common goods may correspond to commodities required for metabolic service functions that benefit all cell types, such as respiration, circulation, digestion and detoxification, or to metabolites such as glutamine and oxygen bound to hemoglobin.

Fig. 1a and Fig. 1c show all possible balanced fluxes through the network of Supplementary Fig. 4. The glucose secretion flux to the environment can assume any positive value ($\omega > 0$), and β_l(the emergent glucose consumption bias) can assume any value between $-\frac{1-\omega}{4}$ and $+\frac{1-\omega}{4}$. All of the subsets of balanced flux patterns that are optimal (in terms

of maximal total specific growth rate) lack glucose secretion (i.e., $\omega = 0$) (Fig. 1b and Fig. 1d), but otherwise their flux pattern depends on the relative cost of the two biomasses. For example, for biomass 1 costing more I1 and thereby more glucose than biomass 2 is costing I2, only a single (Supplementary Table 3) optimal flux pattern is found, i.e., Supplementary Fig. 5. This flux pattern corresponds to the one with β_1=0.25 and $\omega = 0$ (β_1 will equal the initial bias in specific growth rate when the two cell types have equal biomass synthesis yields/stoichiometries) in Fig. 1a, i.e., all carbon flowing to biomass 2. If biomass 1 is cheaper than biomass 2 (i.e., b<1), again only a single optimal flux pattern exists, but now the one with β_1=-0.25 and $\omega = 0$ in Fig. 1a, all carbon flowing to biomass 1. In the singular case that biomasses 1 and 2 are equally expensive (i.e., *b*=1 in Fig. 1a leading to Fig. 1b), flux patterns with all values of β are optimal with respect to the maximum total biomass flux, which amounts to 0.5: multiple choices for flux through each pathway are then optimal (Supplementary Table 4). In all solutions the total biomass synthesis, the I1 to X flux and the I2 to Y flux equaled 0.5/*ts*, 0.25/*ts*, and 0,25/*ts*, respectively, for glucose influx=1/*ts*. The ratio of the G to I1 flux to the G to I2 flux varied, as did the ratio of the two biomass synthesis fluxes. Except if β was equal to 0, there was no co-existence considering the growth of the system if we consider the death rate for cells; always one cell type would go extinct (Fig. 1d). As we consider the case of a continuous absence of growth bias (β=0) unlikely, we conclude that cross dependence of the two cell types through the common goods X and Y is not sufficient for their stable co-existence at maximal total growth rate.

Next, we show that these conclusions are independent of the stoichiometries at which the two biomasses, X, and Y are synthesized from the metabolic intermediates.

3. Cell types competing for common substrate and interdependent through common goods

3-1. Analytical results for arbitrary stoichiometries of X and Y synthesis

Supplementary Fig. 5 shows the only (see Supplementary Table 3) flux pattern predicted for the case where the two biomasses differ in glucose cost with cell type 1 requiring more. Supplementary Fig. 6 shows one of the multiple solutions obtained when the two biomasses were taken to be equally expensive. We also calculated that there only had cross-dependence and no self-dependence in two cell types, i.e., biomass 1 synthesis only uses Y and biomass 2 synthesis only uses X. The result (not shown here) was similar as what was shown here, just the difference of the total objective values.

Fig. 1b shows the range of steady state ('balanced') flux patterns obtained for the network of Supplementary Fig. 5 and Supplementary Fig. 6, where β may assume any value between -0.25 and +0.25 and ω equals to 0. '$\frac{1}{b}$' represents the number of biomass 1 production with 1 unit of I1 molecular; the corresponding number is taken to equal 1 for the synthesis of biomass 2 from I2. When asking for maximal total biomass synthesis, ω becomes equal to zero. The FVA computations of Supplementary Table 3 show that all possible balanced flux patterns are indicated in Fig. 1b by allowing β to assume any value between -0.25 (only synthesis of cell type 1) and +0.25 (cell type 2 only). Supplementary Fig. 7a may be used to prove this for a slightly more general case, in which the pathway synthesizing common good X delivers m molecules of X and 1 molecule of CO_2, whereas the pathway synthesizing common good Y yields n molecules of Y and 1 molecule of CO_2 (The proof is also valid for the cases where metabolism is extended by balanced synthesis and degradation of biomass (Supplementary Fig. 7c)). Without losing generality, the glucose influx is assumed to equal 1 C-mol per time unit. Allowing the glucose assimilation fluxes by biomass 1 and biomass 2 to

differ by twice the glucose 'bias' β_I, the flux balance around glucose requires the fluxes to I1 and I2 to equal $\frac{1-\omega}{2} - \beta_I$ and $\frac{1-\omega}{2} + \beta_I$, respectively (Supplementary Fig. 7a). Again, without essential loss of generality the reactions towards the intermediates both consume 1 molecule of X and Y each. This leads to total consumption fluxes of both X and Y of $1 - \omega$. The flux balance around X and Y thereby require the fluxes from I1 and I2, to CO_2 to equal $\frac{1-\omega}{m}$ and $\frac{1-\omega}{n}$, respectively. These leaves $\frac{(1-\omega)\cdot(m-2)}{2\cdot m} - \beta_I$ and $\frac{(1-\omega)\cdot(n-2)}{2\cdot n} + \beta_I$ for the fluxes towards biomass of type 1 and biomass of type 2, respectively. Total biomass production flux is described by equation (1).

$$biomass1 + biomass2 = \left(\frac{1-\omega}{2}\right) \cdot \left(\frac{m-2}{m\cdot b} + \frac{n-2}{n}\right) + \frac{b-1}{b} \cdot \beta_1 \quad (1)$$

Maximization of total biomass production makes the by-product flux disappear (i.e., $\omega = 0$) and, if b<1 i.e., biomass 1 cheaper than biomass 2, to β_I being as strongly negative as possible, i.e., (Supplementary Fig. 7c):

$$\beta_{optimal\ if\ b<1} = \frac{m-2}{2\cdot m} - \frac{b}{2} \quad (2)$$

$$biomass1_{optimal\ if\ b<1} = \frac{1}{2} \quad (3)$$

and

$$biomass2_{optimal\ if\ b<1} = 0 \quad (4)$$

If biomass 1 is more expensive than biomass 2:

$$\beta_{optimal\ if\ b>1} = \frac{1}{n} \quad (5)$$

and

$$biomass1_{optimal\ if\ b>1} = 0 \quad (6)$$

$$biomass2_{optimal\ if\ b>1} = \frac{1}{2} \quad (7)$$

If both biomasses are equally expensive $\beta_{optimal\ if\ b=1} = any\ value\ between -0.25\ and + 0.25$

and

$$biomass1_{optimal\ if\ b=1} = \frac{m-2}{2 \cdot m} - \beta \tag{8}$$

$$biomass2_{optimal\ if\ b=1} = \frac{n-2}{2 \cdot n} + \beta \tag{9}$$

with their sum

$$(biomass1 + biomass2)_{maximal\ if\ b=1} = \frac{m-2}{2 \cdot m} + \frac{n-2}{2 \cdot n} \tag{10}$$

Supplementary Fig. 6 and Supplementary Fig. 8 are examples of this equally expensive case. If we consider the growth of the system, biomass 1 should be equal to biomass 2. Then, we could find only one co-existence situation (Supplementary Fig. 8):

$$\beta_{co-existence} = \frac{1}{2} \cdot \frac{m-n}{m \cdot n} \tag{11}$$

As we consider this case ($\beta = \frac{1}{2} \cdot \frac{m-n}{m \cdot n}$) highly unlikely always, we conclude that cross dependence of the two cell types though the common goods X and Y is not sufficient for their stable co-existence.

3-2. Growth FBA including cell death

We had anticipated that less synthesis of the more ‘expensive’ biomass (biomass 1 in Supplementary Fig. 5) might lead to a stable co-existence between cell types. After all, reduction of biomass 1 synthesis should make more glucose available thereby stabilizing the cell system. However, our anticipation was wrong: no reduction in biomass synthesis or X synthesis over time was observed in our computations (particularly those in Supplementary Fig. 7b and Fig. 1b for $\beta \neq 0$). This might reflect the limitation of FBA that it delivers steady state fluxes. The integration of biomass synthesis flux revealed a continuous increase in biomass 1 concentration for all β's between -0.25 and +0.25, contrary to our expectations.

In the steady state predicted by FBA, X was still produced at some 25% of the constant glucose influx rate, i.e., at a rate of 0.25 moles/ts.

Apparently, the standard FBA that we had used did not model the situation we had anticipated. Indeed, the FBA of Supplementary Fig. 5, Supplementary Fig. 6 and Supplementary Fig. 8 was calculating the situation of a container supplied with glucose at a constant rate in which both cell types grew without death and the newborn cells exited the container, neither synthesizing nor consuming X nor Y. Standard FBA did not address the situation we had in mind (Fig. 1c-1d and Supplementary Fig. 7c-7d), in which the newborn cells remained in the system (which was to represent a multicellular organism) and contributed fully to metabolism and further growth. Neither does it address the situation of the adult organism in which death of existing cells due to damage or apoptosis is balanced by the generation of new cells. Standard FBA produces flux balance around metabolites, but not around cell densities.

4. Preset exponential growth FBA for two cell types with capacity limitations

We first effected the tendency of the two cell types to grow exponentially by presetting exponential growth equations, as detailed in the Supplementary Methods section. As shown in Supplementary Fig. 9, if cell type 2 had a higher inherent specific growth rate, it always outgrew the cell type 1: still no stable coexistence developed. Paradoxically, as cell type 1 disappeared and with it the capacity to synthesize the common good X, cell type 2 continued to grow even though a shortage of X should arise. The required capacity for X synthesis, i.e., the flux per unit cell 1, increased to infinity (Supplementary Figs. 9d-f).

We then instated a maximum capacity by giving the X synthesis reaction a flux bound of three times the concentration of cell type 1. Supplementary Fig. 10 shows the consequences of this limitation of metabolic capacities for the preset growth model. Initially,

Biomass 2 again increased whilst Biomass 1 decreased and with it the upper bound for X synthesis. After t=12 month (for β(0)=0.05, Supplementary Fig. 10a), the upper bound for X synthesis became lower than what was required, Biomass 2 abruptly decreased with time and the decrease with time of Biomass 1 accelerated. Total biomass thereby also decreased with time, ultimately to zero. For higher initial growth biases in favor of cell type 2, e.g., β(0)=0.1 (Supplementary Fig. 10b) or β(0)=0.2 (Supplementary Fig. 10c), the system behaved similarly, the transition happening earlier.

5. Kinetic growth model for two cell types

With our modified FBA we were still unable to predict that two cell types with different growth tendencies (relative to their death rates) could stably coexist when both depended on common goods they produced. It only shows that they should if they both wanted to have such a maximal growth yield. FBA is an optimum-driven procedure; however, and our FBA analysis was limited to states with maximal total growth rate. The assumed desire of both cell types to exhibit a flux pattern (and to engage in the corresponding genome expression) that made total growth rate (or rather yield, as glucose influx was kept fixed) maximal might derive from evolutionary optimization. However, that same flux pattern may not materialize in a practice that is not the result of the same evolutionary optimization.

Kinetic models are devoid of such optimization requirements and we therefore examined whether a kinetic model of the same system would produce stable co-existence of the two cell types. We therefore modelled the network of Fig. 1d, using irreversible, mass action rate equations. We introduced three growth biases to calculate the biomasses for the two cell types (Supplementary Fig. 13). Our results show that the cell type with lower growth rate disappears first, followed by the other one due to the limitation of the common good

produced by the former cell type. The kinetic model analysis seems to predict the same as what the FBA calculations did, and no coexistence could be established.

We conclude that the failure to produce stable coexistence is not due to the optimization criterion used by the FBA. Rather it must have been due to the interaction between the two cell types not being of the right type or of sufficient strength.

6. Two cell types with competition and common goods; generic considerations

Why is this? We return to the requirement of steady state around cell types 1 and 2:

$$\text{Biomass1}(t - ts) = \frac{b_1(t-ts)}{k_D} \qquad (12)$$

and the same for cell type 2. Consequently,

$$\frac{b_2(t-ts)}{\text{Biomass2}(t-ts)} = k_D \qquad (13)$$

That is: for stable coexistence the specific growth rates (hence the cell cycle times) of the two cell types need to be the same modulo their death rates, essentially requiring the growth bias precisely to compensate for any inherent death bias. Otherwise there can be no stability in this type of stepwise (or exponential) FBA model. But such a model would require cells to keep track of their own density and adjust their specific growth rate to that density, which is an important, yet highly specific type of growth regulation to which we shall return further below. We conclude that some additional regulation is necessary to reach such stability of a multicellular system. Could that regulation be provided by the common goods structure and the maximum metabolic capacities of the two cell types?

Both Biomass1 and b_1 decrease with time and b_2 and Biomass2 increase with time. For Biomass1 this means that:

$$Biomass1(t - ts) > \frac{b_1(t-ts)}{k_D} \qquad (14)$$

We shall now consider the issue that cell type 1 may have a limited capacity to produce X, which corresponds to a maximum flux per unit biomass and which we shall denote by $V_{max.Xsynthesis}$. At some point in the downward curriculum of $B_{1,s}$, it will reach this critical value:

$$B_{1.critical} = \frac{0.25}{V_{max.Xsynthesis}} \quad (15)$$

Should Biomass1 continue to decrease then the production flux of X will drop below the 4 times 0.25/*ts* necessary to sustain a production of 0.5 C-mole of biomass per *ts*, implying that the concentration of X should decrease. Depending on the kinetics of the dependence of the biomass fluxes on the level of X, b_1 or b_2, or both should decrease the objective function, as both b_1 and b_2 should decrease due to lack of X. Consequently, the increase with time of cell type 2 number should become smaller and the decrease of cell type 1 stronger, thereby aggravating the situation. This process would continue until cell type 2 would begin to decrease thereby making way for an increase in b_1 bringing cell type 1 back to its critical level, or the total biomass would decrease as would the need of X. A stable steady state with constant Biomass1 and Biomass2 should ensure, so that:

$$\frac{0.25}{V_{max.Xsynthesis}} \leq Biomass1(t = \infty) = \frac{b_1(t=\infty)}{k_D} \quad (16)$$

And

$$b_2(t = \infty) \leq 0.5 - \frac{0.25 \cdot k_D}{V_{max.Xsynthesis}} \quad (17)$$

And:

$$Biomass2(t = \infty) = \frac{b_2(t=\infty)}{k_D} \leq \frac{0.5}{k_D} - \frac{0.25}{V_{max.Xsynthesis}} \quad (18)$$

This just shows the highest cells number which we can reach for cell type 2, which means the cell number for cell type 2 should be lower than this value if we do not want to reach capacity. But when we use this value as initial values for the cell number, it does not mean the number of cell type 1 and type 2 will not change because the dFBA calculation does not

consider the capacity problem (i.e., the model will not increase the biomass synthesis of cell type 1 if their cell number is not enough for common good production) and biomass synthesis rate for both cell types changes over time. But if we calculate them by using biomass synthesis equation in steady state (the increased biomass equals to their death part) at the beginning, the cell number will not change. We could get a steady state by using any bias ($\beta \neq 0$). The ratio of the growth rates and death rate of the two cell types should follow the equations (19) and (20).

$$b_1(\beta, t) = f(\beta, t) \cdot \mu_1 \cdot Biomass1(t) \quad (19)$$

$$b_2(\beta, t) = f(\beta, t) \cdot \mu_2 \cdot Biomass2(t) \quad (20)$$

so that:

$$\frac{b_1(\beta,t)}{b_2(\beta,t)} = \frac{\mu_1}{\mu_2} \cdot \frac{Biomass1(t)}{Biomass2(t)} = \frac{\mu_1}{\mu_2} \cdot \frac{b_1(\beta,t)}{b_2(\beta,t)} \cdot \frac{k_{D2}}{k_{D1}} \quad (21)$$

This requires that for steady state:

$$\frac{\mu_1}{k_{D1}} = \frac{\mu_2}{k_{D2}} \quad (22)$$

which again corresponds to the requirement that the growth bias precisely compensates for the death rate bias. For above cases (i.e., the equation (22)), we choose the same value for k_{d1} and k_{d2}, so only steady state is $\mu_1 = \mu_2$ which β equals to 0 with same death rate (i.e., $k_{D1} = k_{D2} = 0.5/ts$). Trying the equation $\frac{\mu_1}{k_{D1}} = \frac{\mu_2}{k_{D2}}$ for β with value of 0.05, 0.1, and 0.2, we could find the steady state situation even if the initial growth bias is not equal to zero (Supplementary Fig. 14). But for this situation, it may not be realistic because we make a higher growth rate for one cell type and also with a high death rate, which will lead to less or no effective on increasing cell number.

7. Regulation in Preset-exponential growth and kinetic growth model of two cell types

7-1. Preset-exponential growth FBA with capacity limits and cross-regulation does not lead to stability either

Now we introduce direct cross-regulation between the two cell types. The biomass synthesis rate of cell type 1 ($b_{1,e,r\ ub}(t)$) will now be considered to be positively regulated (ε>0) by the number of cells of type 2, as represented by a factor $\left(B_{2,e,r}(t)\right)^{\varepsilon}$ in its numerator (the negative dependence through its denominator continues to reflect the required Carbon flux balance, *i.e.,* the competition for the glucose). The mechanism of this regulation might be a dependence of the expression level of an enzyme with much flux control on the specific growth rate of cell type 1. This dependence could involve gene expression regulation by a signal transduction route starting with a plasma membrane receptor addressed by a growth factor secreted by cell type 2 (Supplementary Methods). We explored the effect of different regulatory strengths (i.e., ε=0.5 or 1). Supplementary Fig. 15 showed that although this regulation postponed the time at which the system collapsed, it did not establish coexistence.

7-2. Regulation also produces stability in the kinetic growth model of two cell types

As shown in Supplementary Fig. 16, we found that also in the kinetic model, i.e., in the absence of maximization of total growth rate, regulation from biomass value was effective for various growth biases, but the regulation should combine with regulation for common goods and with production inhibition. However, there may exist other regulation methods.

8. Time variant death rates (or growth rate) leading to coexistence

In order to address the time dependent effect of cell death in the presence of a flux balance, we return to the time independent biomass synthesis flux of cell type 1. Addition of a death process with fixed probability leads to:

$$\frac{dB_1}{dt} = 0.25 - \beta - k_D \cdot B_1 \tag{23}$$

With the analogous equation for cell type 2, equation (23) embodies the flux balance of glucose import with glucose consumption for biomass synthesis. The death rate may herewith be time dependent if the Biomass concentration changes with time. Starting with equal densities for the two cell types, this integrates to:

$$k_D \cdot B_1(t) = 0.25 - \beta - \alpha \cdot e^{-k_D \cdot t} \tag{24}$$

with:

$$\alpha = 0.25 - \beta - k_D \cdot B_1(0) \tag{25}$$

Hence:

$$k_D \cdot B_1(t) = k_D \cdot B_1(0) \cdot e^{-k_D \cdot t} + (0.25 - \beta) \cdot (1 - e^{-k_D \cdot t}) \tag{26}$$

and:

$$k_D \cdot B_2(t) = k_D \cdot B_2(0) \cdot e^{-k_D \cdot t} + (0.25 + \beta) \cdot (1 - e^{-k_D \cdot t}) \tag{27}$$

For the biomass synthesis rate of cell type 1 this implies time dependence:

$$0.25 - \beta = b_1 = \mu_{1.death} \cdot B_1(t) \quad = \frac{\mu_{1.death}}{k_D} \cdot \left(k_D \cdot B_1(0) \cdot e^{-k_D \cdot t} + (0.25 - \beta) \cdot (1 - e^{-k_D \cdot t})\right) \tag{28}$$

$$0.25 + \beta = b_2 = \mu_{2.death} \cdot B_2(t) \quad = \frac{\mu_{2.death}}{k_D} \cdot \left(k_D \cdot B_2(0) \cdot e^{-k_D \cdot t} + (0.25 + \beta) \cdot (1 - e^{-k_D \cdot t})\right) \tag{29}$$

Here, the $\mu_{1.death}$ or the $\mu_{2.death}$ is the specific death rate which satisfy that the biomass synthesis rate equals to the death part all the time. However, as we take β to be time

*in*dependent, this also implies that the specific growth rates of both cell types must be time dependent. For β>0 the specific growth rate of cell type 1 increases with time and that of cell type 2 decreases from an initially higher value until they ultimately become equal to each other as well as to the death rate constant:

$$\mu_{1.death}(t) = k_D \cdot \frac{0.25-\beta}{k_D \cdot B_1(0) \cdot e^{-k_D \cdot t} + (0.25-\beta) \cdot (1-e^{-k_D \cdot t})} \qquad (30)$$

$$\mu_{2.death}(t) = k_D \cdot \frac{0.25+\beta}{k_D \cdot B_2(0) \cdot e^{-k_D \cdot t} + (0.25+\beta) \cdot (1-e^{-k_D \cdot t})} \qquad (31)$$

This change in the specific growth rate with time should be mediated by the time dependence of the substrate concentration necessary to bring about the effect of constant flux ratio of the two biomass fluxes. The relaxation times here are short as compared to the times to be simulated, *i.e.,* $1/k_D$. At times more relevant for the developmental process we shall study (i.e., at $large\ t$) :

$$\mu_{1.death}(large\ t) = \mu_{2.death}(large\ t) = k_D \qquad (32)$$

whilst the Biomass concentration has relaxed to the magnitude:

$$B_1(large\ t) = \frac{0.25-\beta}{k_D} \qquad (33)$$

so that the biomass synthesis rate equals the death rate.

$$0.25 - \beta = b_1(large\ t) = B_1(large\ t) \cdot k_D \qquad (34)$$

At these later times, not only do the glucose fluxes balance but so do the fluxes synthesizing and degrading cell types 1 and 2. Consequently, at the longer, more relevant time scales, the effect of the death processes may just as well be neglected (Supplementary Fig. 17).

9. Kinetic growth model for two social cell types and one mutant (without and with regulation)

We also used kinetic models in Copasi to investigate coexistence among the three cell types, with the third type considered as a tumor cell. Upon removing the regulation from and to the

system, the new cell type resulted in making the system unstable (Supplementary Fig. 25). The outgrowth of the mutant cells occurred even though it had the same (or lower) inherent growth rate (Supplementary Fig. 25b).

Then, we reintroduced the regulation from cell type 2 to type 3. As shown in Supplementary Fig. 26, we found that also in the kinetic model, i.e., in the absence of maximization of total growth rate, regulation from cell type 2 to the mutant cell with transferring the glucose to I3 was effective for various regulation strengths (i.e., $\gamma > 1$). However, the effect of regulation from I3 to common good X may vary depending on the regulation methodology used. We showed the effective regulation here, and it may exist other useful regulation methodologies.

10. Alternative objective functions

We used total biomass synthesis rate as objective function in this study. This objective function led to results that appeared descriptive of the stability of multicellular organisms and instability in the case of oncogenic mutations. Perhaps maximum total growth is the standard mechanism in which nonspecific growth factors stimulate the growth of all cells. In the future alternative objective functions, such as a multi-objective function with varying weights for cell types and metabolites[5], should also be considered. Or, the use of maximum biomass of only one cell type, with proper solutions being obtained through cross regulation and competition by and of the other cell types. In preliminary work we already anticipated on this: When we used synthesis of the fastest growing cell type rather than total biomass synthesis as objective function results were the same (results not shown). When we used synthesis of the slowest growing cell type as objective, there was a difference. In that case stable coexistence could already be obtained with only substrate competition and metabolic (X, Y) cross-dependence (Supplementary Fig. 27a), no additional regulation being required. A mutant of the slowest growing cell type 1 merely lacking the ability to synthesize X, did

not destabilize that system (Supplementary Fig. 28a). However, when the cells also regulated each other more directly and again reached stable coexistence (Supplementary Figs, 27b-d), the mutant lacking regulation again outgrew other cell types and destroyed the coexistence (Supplementary Figs, 28b-d). This shows that the main conclusions of the present paper, (i.e., (i) that cell types with different specific growth rates but competing for common substrate can be brought into coexistence through cross talk and (ii) that asocial cells withdrawing from regulation overgrow and then make the system unstable), are independent of which biomass synthesis rate chosen as objective function.

11. More complex systems with the same methodology

We also employed a more complex system (Recon2.2[6]), to replace the metabolic pathways in cell type 1 and type 2 with. We again assumed the two, now more complex, cell types to compete for a metabolite (M0) which they could interconvert into their respective intermediate metabolite (I1 and I2). The I1 and I2 could produce metabolite X and Y, respectively. Furthermore, they could synthesize metabolite M1 and M2, each serving as essential components for biomass synthesis of cell type 1 and type 2, respectively: we incorporated our methodology into this complex system in a simple way. Should one know more about the details of M0, M1, M2, X and Y for the real cells (i.e., the identity of the competition and interdependent metabolites, and their relationship with biomass synthesis), the relevant parameters are readily adjusted and the competition dynamics across diverse cell populations readily simulated. As illustrated in Supplementary Fig. 29, our findings with the complex system corroborated the results we obtained with the simpler system in that (i) the system's stability is compromised when cells exhibit disparate specific growth rates and (ii) the presence of regulatory mechanisms enables the coexistence of cells, even in scenarios involving such different growth rates.

Supplementary Discussion

In summary, cell competition plays a crucial role in maintaining tissue homeostasis, but when disrupted, it can lead to diseases such as cancer. We utilized Cobra[7,8] and dcFBA calculations to describe the regulation and competition among cells. The perhaps important implication is that cancer may not so much be a disease of enhanced growth rate, as that it is due the tumor cells becoming asocial in the sense of unresponsive to regulatory factors. Thus, therapies aimed at reinforcing the cells' cross-regulation, possibly through alternative signaling pathways or an activated immune system, may prove more effective in treating cancer. Other physical or chemical treatments may be effective for a while, but sustained and effective treatment is necessary; otherwise the system will be taken over by tumor cells and eventually becomes unstable (supplementary results); there is no one-size-fits-all description, treatment, or prevention of tumor development[9].

Supplementary Methods

1. Standard FBA for two cell types, each with their own substrate supply: linear growth.

In multicellular organisms most cells have specialized in terms of cell types, each of which belongs to a certain tissue. This situation is potentially hazardous as all cells still have the full potential for cell growth. Should each cell type merely operate towards the objective of its own maximum growth rate, they would not waste resources on helping other cell types. But does this situation by itself necessarily lead to an unstable system? This situation was

simulated for two cell types called 'heart cell' and 'lung cell' that each grew on a substrate that was supplied either (Supplementary Fig. 1 and Supplementary Fig. 2) through a common pool for which they could compete or (Supplementary Fig. 3) to each of them separately. We used standard FBA[1] to calculate what should happen if the objective was total biomass production with equal preference for both types of biomass (Objective=b_H+b_L). First, we did the FBA and related FVA[2] calculation for two cell types which competed for glucose and had different growth yields. Then, we made two cell types equal biomass synthesis yield and did the FBA and FVA calculation again to check whether the two cell types could lead to co-existence. We also calculated the case that each cell types have their own glucose supplied with diversity growth yields. For all calculation here, we provided glucose with constant rate of 1/*ts*.

2. Preset-exponential growth FBA for two cell types with cross dependence through common goods, obeying the optimal FBA condition of constant optimal total biomass flux

As the system tends to be developing, we assumed exponential functions of time for the two growth tendencies *f(t),* i.e., the equations are described by equations (35) and (36).

$$b_{1,e,ub}(t)= 0.5\cdot \frac{\mu_1\cdot e^{\frac{\mu_1\cdot t}{\mu_1+\mu_2}}}{\mu_1\cdot e^{\frac{\mu_1\cdot t}{\mu_1+\mu_2}}+\mu_2\cdot e^{\frac{\mu_2\cdot t}{\mu_1+\mu_2}}} \tag{35}$$

$$b_{2,e,ub}(t)= 0.5\cdot \frac{\mu_2\cdot e^{\frac{\mu_2\cdot t}{\mu_1+\mu_2}}}{\mu_1\cdot e^{\frac{\mu_1\cdot t}{\mu_1+\mu_2}}+\mu_2\cdot e^{\frac{\mu_2\cdot t}{\mu_1+\mu_2}}} \tag{36}$$

μ_1=0.25-β and μ_2=0.25+ β are the specific growth rates for cell types 1 and 2 and assumed to be constant. We used the '$\frac{\mu_1}{\mu_1+\mu_2}$' and '$\frac{\mu_2}{\mu_1+\mu_2}$' to normalize the growth rates for both cell types here. The parameter β is the initial growth rate bias in favor of cell type 2. Because the

growth rate is set *a priori* as an exponential function of time we call this the 'preset-exponential FBA' procedure.

A familiar way to make FBA produce maximal flux through a reaction is to give the corresponding reaction an upper bound equal to that flux and all other reactions higher upper bounds. We therefore set the upper bounds for the biomass synthesis reactions equal to the equations (35) and (36), and then carried out FBA for Supplementary Fig. 4 at subsequent time points, separated from each other by ts (a small (infinitesimal) amount of time units) and with glucose efflux $1/ts$. This produced $b_{1,e,FBA}(t)$ and $b_{2,e,FBA}(t)$, which were indeed identical to the upper bounds. Acknowledging that the cells should also be subject to death processes (for which we used a first order process with rate constant k_D (we chose k_D to equal $0.5/ts$)), we calculated the Biomass concentrations for the two cell types at each time point by equations (37) and (38).

$$B_{1,e}(t+ts) = B_{1,e}(t)\cdot(1-ts\cdot k_D)+ts\cdot b_{1,e,FBA}(t) \quad (37)$$

$$B_{2,e}(t+ts) = B_{2,e}(t)\cdot(1-ts\cdot k_D)+ts\cdot b_{2,e,FBA}(t) \quad (38)$$

where ts=0.1 month; $B_1(0)$=0.4 and $B_2(0)$=0.6 for β=0.05; $B_1(0)$=0.3 and $B_2(0)$=0.7 for β=0.1; $B_1(0)$=0.1 and $B_2(0)$=0.9 for β=0.2 (These values were the same for subsequent calculations unless specifically stated). We chose these values because we wanted the whole system to be stable before it becomes unstable (new biomass flux equaling the death flux). The difference between the cell numbers at the beginning was in accordance with their relative growth rates.

3. Kinetic growth model for two cell types

In kinetic model, cell type 1 utilizes glucose to produce intermediate metabolite I1 which is further converted into biomass or a common good X. This reaction requires the consumption of common goods X and Y with stoichiometries of 1 and is motivated by the number of cells

of type 1. Simultaneously, cell type 2 also uses glucose to produce intermediate metabolite I2, which contributes to its biomass production or common good Y production. This process is driven by the number of cell type 2. The production of biomass1 or X is activated by the number of cell type 1, whilst the cell type 2 number could induce biomass2 production or Y production. The details of the reaction equations and their rate laws are shown in Supplementary Table 5. The kinetic model is provided at Github. B1(0)=0.5 and $B_2(0)$=0.5; Glc(0)=I1(0)= I2(0)=X(0)=Y(0)=1 (These values were the same for subsequent kinetic calculations unless specifically stated). The kinetic modelling did not depend on any objective function.

4 Regulation in preset-exponential growth FBA

We also introduced cross regulation between cell types, at a strength again indicated by the parameter 'ε'[3] in the 'preset-exponential growth' FBA model as described by equations (39) and (40).

$$b_{1,e,r\ ub}(t) = 0.5 \cdot \frac{(0.25-\beta)\cdot e^{\frac{(0.25-\beta)\cdot t}{0.5}} \cdot \left(B_{2,e,r}(t)\right)^{\varepsilon}}{(0.25-\beta)\cdot e^{\frac{(0.25-\beta)\cdot t}{0.5}} \cdot \left(B_{2,e,r}(t)\right)^{\varepsilon} + (0.25+\beta)\cdot e^{\frac{(0.25+\beta)\cdot t}{0.5}} \cdot \left(B_{1,e,r}(t)\right)^{\varepsilon}} \quad (39)$$

$$b_{2,e,r,ub}(t) = 0.5 \cdot \frac{(0.25+\beta)\cdot e^{\frac{(0.25+\beta)\cdot t}{0.5}} \cdot \left(B_{1,e,r}(t)\right)^{\varepsilon}}{(0.25-\beta)\cdot e^{\frac{(0.25-\beta)\cdot t}{0.5}} \cdot \left(B_{2,e,r}(t)\right)^{\varepsilon} + (0.25+\beta)\cdot e^{\frac{(0.25+\beta)\cdot t}{0.5}} \cdot \left(B_{1,e,r}(t)\right)^{\varepsilon}} \quad (40)$$

As before we used these equations to set the upper bounds for the biomass synthesis reactions in the metabolic scheme and then carried out FBA with the total biomass synthesis as objective. The resulting biomass synthesis for both cell types were again used to calculate the cell levels for the next time point as described in the equations (37) and (38). As these equations contain a predefined exponential growth tendency, we call this procedure 'regulated preset-exponential growth FBA'.

5. Regulation in the kinetic growth model for two cell types

For the kinetic growth model, we incorporated the regulation power by the parameter ‘θ’ from glucose to I1 and I2, and also the regulation power by the parameter ‘ρ’ for producing common goods by equations (41), (42), (43) and (44).

$$v_{Glc-I1} = k_{R1} \cdot [Glc(t)] \cdot [X(t)] \cdot [\mathrm{Y(t)}] \cdot [B_1(t)] \cdot [B_2(t)]^{\theta} \quad (41)$$

$$v_{Glc-I2} = k_{R5} \cdot [Glc(t)] \cdot [X(t)] \cdot [\mathrm{Y(t)}] \cdot [B_1(t)] \cdot [B_2(t)]^{\theta} \quad (42)$$

$$v_{I1-X} = k_{R3} \cdot [I_1(t)] \cdot [B_1(t)] \cdot [B_2(t)]^{\rho}/(1 + \frac{[X(t)]}{10}) \quad (43)$$

$$v_{I2-Y} = k_{R7} \cdot [I_2(t)] \cdot [B_2(t)] \cdot [B_1(t)]^{\rho}/(1 + \frac{[\mathrm{Y(t)}]}{10}) \quad (44)$$

where v_{Glc-I1} and v_{Glc-I2} are the rates from glucose to intermediate metabolites I1 and I2, respectively. v_{I1-X} and v_{I2-Y} are the rates from intermediate metabolites I1 and I2 to metabolite X and Y, whilst k_{R1} and k_{R5} are the parameters for the reactions from glucose to the respective intermediary metabolites, k_{R3} and k_{R7} are the parameters for the respective intermediary metabolites to metabolite X and Y. In the kinetic model, we did not put regulation in the rate equations of the other reactions (i.e., they were the same as the rate laws in Supplementary Table 5). $Glc(t)$, $X(t)$, $Y(t)$, $I_1(t)$, $I_2(t)$, $B_1(t)$, and $B_2(t)$ are the concentration of glucose, X, Y, cell type 1 and type 2, respectively, at time ‘t’. After using the regulated function for glucose consumption, we ran the time course algorithm of Copasi[4] for the model and calculated the relationship between the cell numbers and time.

6. kinetic growth model for three cell types

After calculating the kinetic growth model with two cell types, we added another cell type to the system, which could be simulated as a mutant of cell type 1. Again, the new cell type (type 3) could produce common good X and use common goods X and Y to convert glucose to its intermediate metabolite I3, but it lost the regulation by and to cell type 2 (Cell type 3 in

this case is more similar as tumor cell). The rate constant values and rate law for equations of cell type 3 are same as cell type 1 only without regulation from type 2. The original concentration values for metabolites in the system were taken from the balance in two cell types system: Glc(0)=0.253; I1(0)=0.125; I2(0)=0.083; X(0)=0.291; Y(0)=2.682; $B_1(0)=0.375$; $B_2(0)=0.625$; b1(0)=0.187 and b2(0)=0.313 (These values are gotten from the coexistence in two cell types system). And cell type 3 is 0.02 ($B_3(0)=0.02$), I3 is 0.02 (I3(0)=0.02). After building the model and setting the parameters, we ran the time course algorithm in Copasi for the model and calculated the co-existence of the system.

6-1 Regulation for kinetic model of three cell types

We could not find a coexistence for three cell types when we added a mutant of cell type 1 (cell type 3) into the system in kinetic model. As cell type 3 simulated as a tumor cell, it may not lose all regulation from the system. In actual situations, the transformed cell needs support from normal cells, but itself may not support the normal cells in any way. Therefore, we considered to put the regulation from cell type 2 back to type 3 with the reaction transferring glucose to I3. The regulated equation is shown in equation (45).

$$v_{Glc-I3} = k_{R1} \cdot [Glc(t)] \cdot [X(t)] \cdot [\mathrm{Y(t)}] \cdot [B_3(t)] \cdot [B_2(t)]^{\gamma} \qquad (45)$$

where 'γ' is the regulation power (elasticity) from cell type 2 to type 3. Again, we ran the time course algorithm in Copasi and did the calculation for the system after setting the model.

Supplementary References

[1] Orth JD, Thiele I, Palsson BØ. What is flux balance analysis?. *Nat Biotechnol.* 28(3): 245-248. https://doi.org/10.1038/nbt.1614 (2010).

[2] Burgard, A. P., Vaidyaraman, S., & Maranas, C. D. Minimal reaction sets for *Escherichia coli* metabolism under different growth requirements and uptake environments. *Biotechnology progress*, *17*(5), 791–797. https://doi.org/10.1021/bp0100880 (2001).

[3] Burns, J. A., et al. 'Control analysis of metabolic systems', Trends in Biochemical Sciences, 10: 16-16. (1985).

[4] Hoops, S., et al. COPASI--a COmplex PAthway SImulator. *Bioinformatics (Oxford, England)*, *22*(24), 3067–3074. https://doi.org/10.1093/bioinformatics/btl485 (2006).

[5] Liu, Y., & Westerhoff, H. V. Competitive, multi-objective, and compartmented Flux Balance Analysis for addressing tissue-specific inborn errors of metabolism. Journal of inherited metabolic disease. https://doi.org/10.1002/jimd.12603 (2023)

[6] Swainston, N., Smallbone, K., Hefzi, H., Dobson, P. D., Brewer, J., et al. Recon 2.2: from reconstruction to model of human metabolism. Metabolomics: Official journal of the Metabolomic Society, 12, 109. https://doi.org/10.1007/s11306-016-1051-4 (2016).

[7] Heirendt L, Arreckx S, Pfau T, et al. Creation and analysis of biochemical constraint-based models using the COBRA Toolbox v.3.0. *Nat Protoc.* 14(3): 639-702. https://doi.org/10.1038/s41596-018-0098-2 (2019).

[8] Ebrahim A, Lerman JA, Palsson BO, Hyduke DR. COBRApy: COnstraints-Based Reconstruction and Analysis for Python. *BMC Syst Biol.* 7: 74. https://doi.org/10.1186/1752-0509-7-74 (2013).

[9]: Kulavi S., Ghosh C., Saha M., Chatterjee S. One size does not fit all: An overview of personalized treatment in cancer. *JPRI*, 87–103. https://doi.org/10.9734/jpri/2021/v33i28a31513 (2021).
